# Genome resequencing reveals the evolutionary history of garlic reproduction traits

**DOI:** 10.1093/hr/uhad208

**Published:** 2023-10-17

**Authors:** Haiping Wang, Einat Shemesh-Mayer, Jiangjiang Zhang, Song Gao, Zheng Zeng, Zemao Yang, Xueyu Zhang, Huixia Jia, Yanzhou Wang, Jiangping Song, Xiaohui Zhang, Wenlong Yang, Qiaoyun He, Amir Sherman, Lin Li, Rina Kamenetsky, Touming Liu

**Affiliations:** State Key Laboratory of Vegetable Biobreeding, Institute of Vegetables and Flowers, Chinese Academy of Agricultural Sciences, Beijing, China; Institute of Plant Sciences, Agricultural Research Organization—The Volcani Institute, Rishon LeZion, Israel; Institute of Bast Fiber Crops, Chinese Academy of Agricultural Sciences, Changsha, China; College of Horticulture and Landscape Architecture, Yangzhou University, Yangzhou, China; Institute of Bast Fiber Crops, Chinese Academy of Agricultural Sciences, Changsha, China; Institute of Bast Fiber Crops, Chinese Academy of Agricultural Sciences, Changsha, China; Institute of Bast Fiber Crops, Chinese Academy of Agricultural Sciences, Changsha, China; State Key Laboratory of Vegetable Biobreeding, Institute of Vegetables and Flowers, Chinese Academy of Agricultural Sciences, Beijing, China; Institute of Bast Fiber Crops, Chinese Academy of Agricultural Sciences, Changsha, China; Industrial Research Institute of garlic (IBFC-Jinxiang), Jinxiang, China; State Key Laboratory of Vegetable Biobreeding, Institute of Vegetables and Flowers, Chinese Academy of Agricultural Sciences, Beijing, China; State Key Laboratory of Vegetable Biobreeding, Institute of Vegetables and Flowers, Chinese Academy of Agricultural Sciences, Beijing, China; State Key Laboratory of Vegetable Biobreeding, Institute of Vegetables and Flowers, Chinese Academy of Agricultural Sciences, Beijing, China; Institute of Bast Fiber Crops, Chinese Academy of Agricultural Sciences, Changsha, China; Institute of Plant Sciences, Agricultural Research Organization—The Volcani Institute, Rishon LeZion, Israel; College of Plant Science and Technology, Huazhong Agricultural University, Wuhan, China; Institute of Plant Sciences, Agricultural Research Organization—The Volcani Institute, Rishon LeZion, Israel; College of Horticulture and Landscape Architecture, Yangzhou University, Yangzhou, China; Industrial Research Institute of garlic (IBFC-Jinxiang), Jinxiang, China

## Abstract

The propagation of cultivated garlic relies on vegetative cloves, thus flowers become non-essential for reproduction in this species, driving the evolution of reproductive feature-derived traits. To obtain insights into the evolutionary alteration of reproductive traits in the clonally propagated garlic, the evolutionary histories of two main reproduction-related traits, bolting and flower differentiation, were explored by genome analyses using 134 accessions displaying wide diversity in these two traits. Resequencing identified 272.8 million variations in the garlic genome, 198.0 million of which represent novel variants. Population analysis identified five garlic groups that have evolved into two clades. Gene expression, single-cell transcriptome sequencing, and genome-wide trait association analyses have identified numerous candidates that correlate with reproductive transition and flower development, some of which display distinct selection signatures. Selective forces acting on the B-box zinc finger protein-encoding *Asa2G00291.1*, the global transcription factor group E protein-encoding *Asa5G01527.1*, and *VERNALIZATION INSENSITIVE 3*-like *Asa3G03399.1* appear to be representative of the evolution of garlic bolting. Plenty of novel genomic variations and trait-related candidates represent valuable resources for biological studies of garlic. Numerous selective signatures from genes associated with the two chosen reproductive traits provide important insights into the evolutionary history of reproduction in this clonally propagated crop.

## Introduction

Our ability to investigate crop domestication and evolution has greatly improved with the development of high-throughput sequencing and the identification of selection signatures in genes associated with favorable traits. Numerous studies have revealed that crops gain favorable characteristics at the population level [1, 2]. However, the mechanisms underlying the loss of fertility and degeneration of reproductive organs in clonally propagated crops during their evolution and domestication remain unclear.

Garlic (*Allium sativum* L.) has been an important vegetable and medicinal crop for over 10 000 years [3]. Currently, this crop is known only in cultivation, but its wild relatives are found in Central Asia. Garlic’s ancestor was probably domesticated there and later introduced to the Mediterranean basin, India, China, and then to other world regions [4]. Presumably, early garlic crops produced small bulbs, flowers, and seeds, which are similar to those produced by wild *Allium* species. Following domestication, human selection led to the enlargement of storage organ bulbs, limiting resources for blooming and resulting in sterility [3]. Hence, modern garlic varieties are clonally propagated and further improvements are limited to random mutations or stable epigenetic effects [5, 6]. However, fertile garlic genotypes are still found in Central Asia [7]. In addition, garlic clones from various regions differ in their reproductive traits. For example, either flower initiation and bolting are completely impaired, or small bulblets (topsets) that develop within the inflorescence compete with flowers for nutrients. In some genotypes, when individual flowers succeed in differentiating, seed production is compromised because of male or female sterility, tapetum degeneration, and pollen abortion [7–10].

Generally, *Allium* species possess relatively large genomes compared with other eukaryotes [10], presenting a challenge in exploring the evolutionary history of *Allium* crops. Nevertheless, genome analyses of bunching onions (*Allium fistulosum*) have shed light on flavor formation during its domestication history by identifying the selection signatures of genes involved in alliin biosynthesis [10]. In garlic, variations in 606 accession sequences were detected by genotyping-via-sequencing, which revealed that genes for genomic selection regions between complete-bolting and non-bolting accessions were enriched in functional terms associated with reproductive growth [11]. In a recent study, whole-genome resequencing was performed for 84 accessions, finding that *A. sativum* experienced a bottleneck during the Younger Dryas period because of a cold environment, resulting in the proposal that this process culminated in plant ability to resist low temperatures [12]. Furthermore, numerous selection signatures in bulb growth-related genes indicate strong selection for increasing bulb size during garlic evolution and domestication history [12].

With the evolution of bulb-based vegetative propagation, sexual reproduction seems to become unessential for this crop, permitting traits related to sexual reproduction to be investigated. Although evolutionary analyses of garlic have been conducted using genome-wide variations from 84 accessions in previous research [12], the evolution of reproduction-related traits has not been assessed, because all of these sequenced accessions lacked flowering ability. To explore the evolution of sexual reproduction in this *Allium* crop, we resequenced an additional 50 garlic accessions, including 14 genotypes with flowering ability. These 50 accessions, together with the 84 accessions previously reported by Li *et al*. [12], were used to analyze the evolution of the major reproduction-related traits, bolting ability, and flower differentiation. Candidate genes associated with reproductive traits and their selective signatures will provide insights into the degeneration of sexual reproduction in clonally propagated garlic.

## Results

### Genomic variation

Genome resequencing of the 50 garlic genotypes generated ~53.9 billion clean reads (8.08 Tb total sequence length) (Supplementary Data Table S1). Approximately 99.22% of the sequence reads were successfully aligned to the garlic reference genome (PRJNA606385 in NCBI [13]), with an average depth of 9.37-fold coverage (Supplementary Data Table S1). Through this resequencing, we identified 250 934 179 SNPs and 21 933 343 small insertions or deletions (indels; ≤5 bp) (Supplementary Data Tables S2 and S3). A total of 4 960 349 (1.98%) SNPs and 676 958 (3.09%) indels were located in coding regions. Among these, 386 512 non-synonymous SNPs and 32 950 indels potentially resulted in either a frameshift or a new stop codon within the encoding sequences. Compared with the genomic variations previously identified within 84 accessions [12], many more variations (approximately 2.56-fold indels and 2.08-fold SNPs) were detected in the present study, of which, 182 131 135 SNPs and 15 907 046 indels were identified as novel. Therefore, these newly sequenced genotypes add valuable new genetic data and provide an important resource for studies on flowering biology and breeding of this economically important crop.

### Population structure and diversity

**Figure 1:**
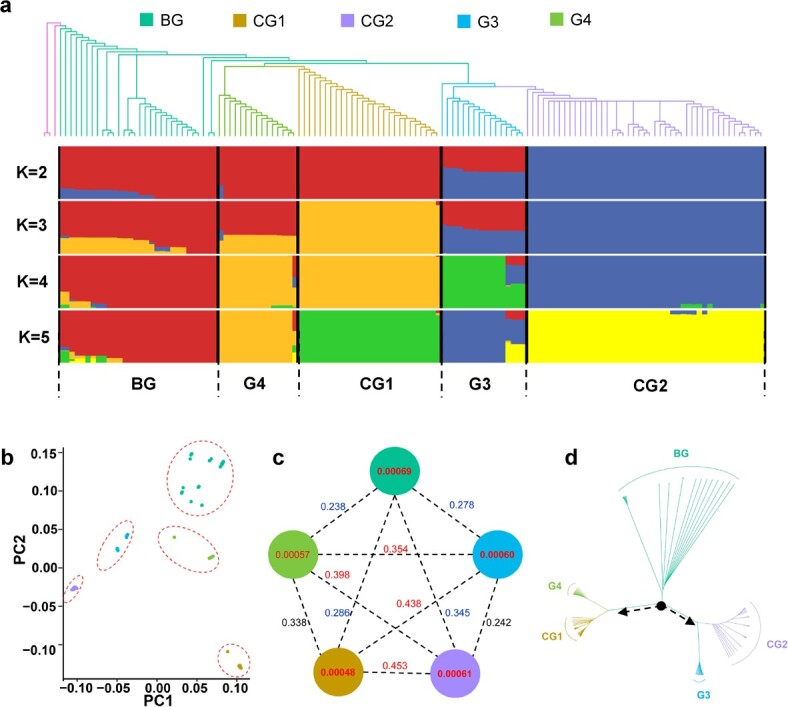
Population structure and genetic diversity of garlic accessions. **a** Phylogenetic tree and model-based clustering analysis of 134 accessions. The rooted tree was constructed using the maximum likelihood method. Model-based clustering analysis with different cluster numbers (*k* = 2–5). The y-axis quantifies cluster membership and the x-axis lists the different accessions. The orders and positions of these accessions on the x-axis are consistent with those presented in the neighbor-joining tree. Three *A. ampeloprasum* accessions were used as the outgroup species. **b** Principal component analysis plots of the first two components of the 134 accessions. **c** Nucleotide diversity (π) and population divergence (*F*_ST_) across the five groups. The value in each circle represents an estimation of nucleotide diversity for each group, and values on each line indicate pairwise population divergences between groups. **d** Maximum likelihood phylogenetic tree for 134 accessions based on SNPs. Two different evolutionary clades derived from the BG were identified.

We performed a population analysis using 134 accessions, including the 50 sequenced in this study and 84 previously reported accessions, that represented a wide collection from 30 countries (Supplementary Data Table S4). Genetic relationships among accessions were assessed using maximum likelihood phylogeny rooted in *Allium ampeloprasum*, admixture, and principal component analysis. Our analyses showed strong clustering of the studied accessions into five groups (Fig. 1a and b); i.e. a basal group (BG) including 28 accessions, and four other groups clustered in accordance with previously reported divisions [12], namely, cultivated group 1 (CG1; 27 accessions), cultivated group 2 (CG2; 46 accessions), group 3 (G3; 16 accessions), and group 4 (G4, referred to as OG in a previous study; 17 accessions). Notably, all 14 flowering accessions belonged to the BG. The genetic diversity among the BG was large (θπ = 0.00069) relative to the other four groups (θπ value ranged from 0.00048 to 0.00061) (Fig. 1c). To estimate genomic divergence among the five populations, we calculated the pairwise genome-wide fixation index (*F*_ST_) value. *F*_ST_ values ranged from 0.238 to 0.453 (Fig. 1c), indicating large genomic divergence among garlic populations, consistent with a recent study [12].

### Evolutionary analysis of garlic population

**Figure 2:**
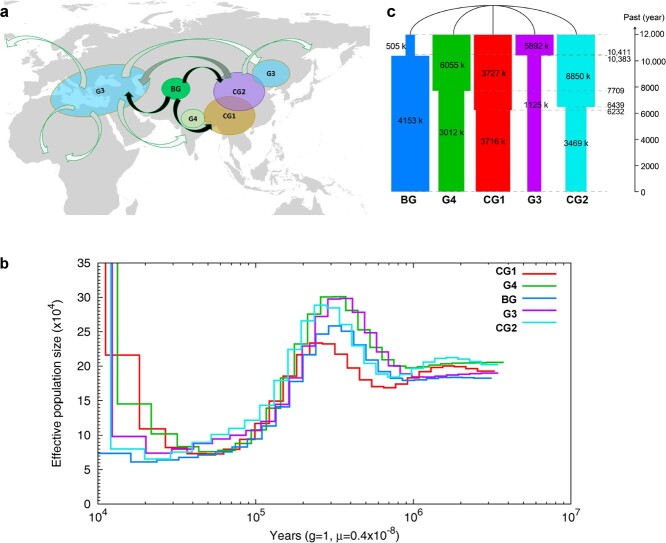
Evolutionary history of garlic. **a** A putative domestication route of the garlic crop. The basal group (BG) in Central Asia contains numerous genotypes with flowering ability, and develops in two directions: to the east, forming co-evolved G4 and CG1 groups and the separate CG2 group; and to the west, forming the G3 group (black arrows). Secondary domestication included germplasm exchange between east (CG1, CG2) and west (G3) (gray arrows). Later, the domestication and introduction processes were intensified by human travel and agricultural development on a global scale (white arrows). **b** Demographic history of five garlic groups inferred from the estimation of the historical effective population size *N*_e_ using the PSMC method. **c** Graphical summary of the best-fitting demographic model inferred by fastsimcoal2. Widths show relative *N*_e_.

Phylogenetic analysis separated the accessions into BG and two evolutionary clades. CG1 and G4 clustered in one clade and CG2 and G3 clustered into another (Fig. 1d). Additionally, we observed that the genome divergences between groups from different evolutionary clades were larger (*F*_ST_ > 0.354) than those between groups and the BG (*F*_ST_ < 0.345), especially among the genomes within cultivated CG1 and CG2, with an *F*_ST_ value of 0.453 (Fig. 1c). These results suggest that the garlic population might have evolved following two different routes (i.e. BG–G4–CG1 and BG–G3–CG2), supporting the previous proposal that CG1 and CG2 were domesticated independently [12]. Additionally, we observed that the *F*_ST_ values between BG and G4/G3 were 0.238 and 0.278, lower than those between BG and CG1/CG2 (0.286 and 0.345, respectively; Fig. 1c), indicating a larger genomic divergence between BG and CG1/CG2 than between BG and G4/G3. Combining these findings with the geographical locations of accessions and the results of a previous study [14], we speculated possible migration routes of garlic as follows: initial domestication developed in two directions: to the east, forming co-evolved G4 and CG1 groups and the separate CG2 group, and to the west, forming the G3 group; subsequent secondary domestication included germplasm exchange between east (CG1, CG2) and west (G3), then domestication and introduction processes were intensified by human travel and agricultural development on a global scale (Fig. 2a).

We further investigated the demographic history of garlic by estimating its historical effective population size (*N*_e_) using the pairwise sequential Markovian coalescence (PSMC) method. All five garlic groups displayed similar demographic trajectories (Fig. 2b); i.e. the ancestral *N*_e_ of *A. sativum* peaked 200–300 thousand years ago, followed by a continuous decline until the end of the last glacial maximum (~20 000 years before present). Interestingly, after the bottleneck period, the population scales from different evolutionary clades increased at different periods, i.e. CG1 and G4 expanded earlier than CG2 and G3 (Fig. 2b). Because no increase in *N*_e_ was observed in the BG by PSMC analysis, we used coalescent simulations to model the recent demography of garlic. The BG population scale began to expand ~10 000 years ago (Fig. 2c). Notably, all cultivated groups displayed decreases in their effective population scales over the last 10 000 years, especially the two groups of clade G3/CG2, with reductions of 80.9% (G3) and 60.8% (CG2). Garlic was domesticated in Central Asia more than 10 000 years ago [3]. Because domestication frequently causes loss of genetic diversity, we assume that domestication is one potential reason for the decrease in the effective scale of cultivated garlic.

To identify genomic regions associated with garlic domestication, we detected the selective signal of four cultivated groups by comparing with BG, based on three methods: (i) population branch statistics (PBS), (ii) estimation of θπ ratios (π_BG_/π_CG1_, π_BG_/π_CG2_, π_BG_/π_G3_, and π_BG_/π_G4_), and (iii) the test of cross-population composite likelihood ratio (XP-CLR; CG1/CG2/G3/G4 versus BG). Genomic regions with the top 5% estimated values using at least two of the three methods were considered putative selective sweeps. We identified a total of 2291, 1484, 1690, and 1979 putative selective sweeps in CG1, CG2, G3, and G4, respectively, with respective lengths of 520.2, 483.6, 360.8, and 325.3 Mb (Supplementary Data Tables S5–S7). A total of 1739, 1731, 1213, and 898 genes were identified in the respective selective regions of CG1, CG2, G3, and G4 (Supplementary Data Table S8), 600 of which were subjected to common selection in at least two garlic subpopulations. Interestingly, although the genome divergence between CG1 and CG2 (*F*_ST_ = 0.453) was larger than that between G3 and G4 (*F*_ST_ = 0.354), there was a greater overlap of the selective genome between CG1 and CG2 (43.0 Mb, including 135 genes) than between G3 and G4 (18.29 Mb, harboring 60 genes).

### Diversification of bolting performance among garlic populations

Bolting and non-bolting genotypes differed in performance (Supplementary Data S1). Our assessment of the bolting ability of the studied genotypes showed that bolting ratios varied among the five groups (Supplementary Data S2). More than 28% of BG accessions did not produce flower stems. The proportions of non-bolting accessions in G3 and G4 reached 47 and 87%, respectively, whereas those in CG1 and CG2 only reached 3.7 and 8.9%, respectively. These results indicate that bolting ability underwent selection during garlic domestication. To investigate the selection signatures potentially related to the evolution/domestication effects on garlic bolting, we explored bolting time-controlling genes in garlic using transcriptomic analysis. A total of 3539 genes showed a change in expression in reproductive stage garlic leaves compared with vegetative growth (Supplementary Data S3; Supplementary Data Table S9). Of these genes, 94, 116, 63, and 47 underwent potential selection in CG1, CG2, G3, and G4, respectively, and 38 showed significant selective signals in at least two garlic groups (Supplementary Data S4).

**Figure 3:**
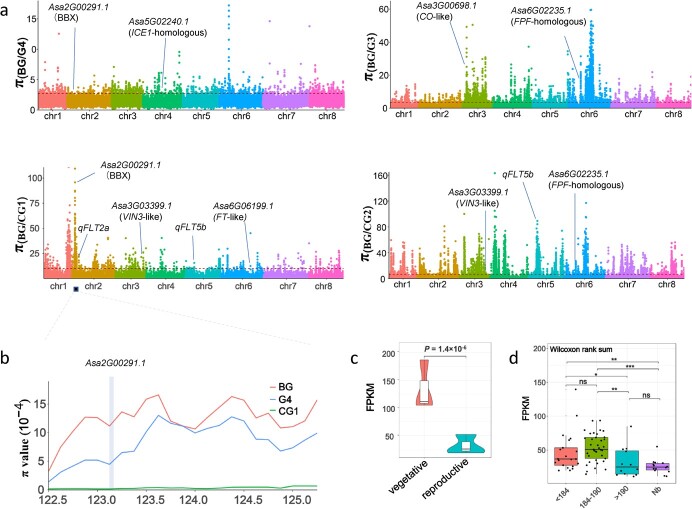
Selection signatures for bolting time in garlic. **a** Genomic regions with top 5% π_BG_/π_G4_ (or π_BG_/π_CG1_, π_BG_/π_CG2_, π_BG_/π_G3_) values. Horizontal dashed lines indicate the genome-wide thresholds of the selection signals from analysis of π ratio values. Candidate genes identified in this study are marked. **b** Distribution of nucleotide diversity (π) of BG, G4, and CG1 accessions in the 122.5- to 125-Mb region. *CO*-like *AsaG00291.1* was located within the sweep. **c** Expression level of *Asa2G00291.1* displayed a significant difference between garlic leaves undergoing vegetative and reproductive growth. **d** Expression differences among accessions with bolting time of <184, 184–190, and > 190 days and accessions without bolting ability (Nb). *, **, and *** indicate respective significance levels at 0.05, 0.01, and 0.001. Bolting time was investigated by counting the days from planting to bolting.

Thirty-four differentially expressed genes (DEGs) that encode homologs of *Arabidopsis* flowering-controlled proteins were identified. These genes constitute a putative regulatory network for controlling garlic bolting (Supplementary Data S5). Of these 34 DEGs, seven were *CO*-like genes and six were *FT*-homolog genes (Supplementary Data S6 and S7). *CO*-like *Asa3G03846.1* and *FT*-like *Asa7G06383.1*, whose expression was significantly increased in garlic leaves at the reproductive stage, were selected for functional analysis. We found that the overexpression of these genes caused early flowering in transgenic *Arabidopsis* (Supplementary Data S8). Notably, the *CO*-like *Asa3G00698.1* and flowering-promoting *FT*-like *Asa6G06199.1* [12] were selected in CG1 and G3, respectively, during garlic evolution/domestication (Fig. 3a, Supplementary Data S9). This result confirms the role of *CO*/*FT*-like genes in garlic bolting and their possible divergent selection during garlic domestication (Fig. 3a).

We also identified a B-box zinc finger protein (BBX)-encoding *Asa2G00291.1* with a distinct selection signature in both CG1 and G4 (Supplementary Data S9). In CG1, *Asa2G00291.1* was located in a ~2.8-Mb-length selective sweep whose diversity loss was great compared with BG (π_BG_/π_CG1_ = 66.9) (Fig. 3b). Only one haplotype could be identified in this group (Supplementary Data S10). These results indicate that *Asa2G00291.1* underwent continuous selections along the evolutionary route of BG–G4–CG1. *Asa2G00291.1* showed downregulated expression in garlic during reproductive growth (Fig. 3c), and an investigation of the correlation between *Asa2G00291.1* transcript abundance and bolting time in 81 accessions (transcriptome data reported by Li *et al*. [12]) revealed a significant negative correlation (correlation coefficient = −0.395, *P* = .000197) (Fig. 3d). Our expression results thus suggest a similar role for this BBX homolog in controlling bolting time in garlic, and its evolution can have potential impacts on bolting time in garlic accessions of the coevolved CG1 and G4.

**Figure 4:**
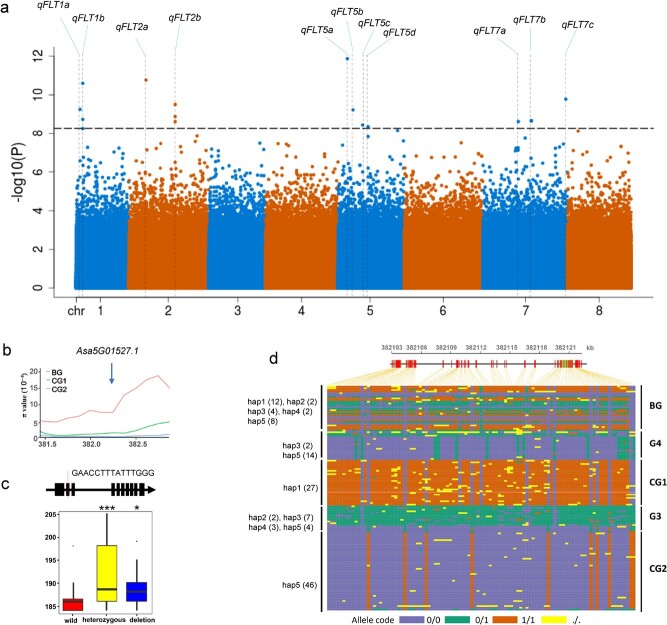
Selective sweeps overlapped with genetic loci associated with bolting time. **a** Whole-genome association analysis of the bolting time trait in 230 garlic accessions reported by Li *et al*. [12]. Drashed lines indicate the associated position with a significant signal (*P* < 5.32 × 10^−9^). **b** Distribution of nucleotide diversity (π) of BG, G4, and CG1 accessions in the region near the candidate *qFLT5b* homolog, *Asa5G01527.1*. The arrow indicates the position of *Asa5G01527.1*. **c** A 15-bp deletion was identified in the second exon of *Asa5G01527.1*. Bolting time of accessions harboring heterozygous and mutant alleles is significantly greater than that of those harboring wild type, with a *P* value of .00064 and .0449, respectively. **d** Haplotypes for *Asa5G01527.1* in 134 accessions. Only one haplotype is identified in CG1 and CG2.

We further explored potential selective genes associated with bolting time by performing genome-wide association analysis using 230 garlic accessions. Fourteen signals from 11 genomic regions were associated with bolting (Fig. 4a). Two and one association regions overlapped with the selective sweeps of CG1 and CG2, respectively (Fig. 3a). Notably, we observed a genomic region (around 381.6–382.7 Mb on chromosome 5) whose nucleotide diversity was markedly reduced both in CG2 (π_BG_/π_CG2_ = 43.4) and CG1 (π_BG_/π_CG1_ = 8.9), compared with BG (Fig. 4b), indicating that this interval underwent common selection in two cultivated groups. An association locus, *qFLT5b*, related to bolting time was located within this selective sweep. This sweep included two genes, *Asa5G01527.1* and *Asa5G01528.1*, but only *Asa5G01527.1* expression showed a negative association with bolting time in a garlic population consisting of 81 accessions (correlation coefficient = −0.225, *P* < .05). *Asa5G01527.1* encodes a putative global transcription factor group E (GTE) protein, which is an ortholog of *Arabidopsis* GTE10 (Supplementary Data S11). We observed a 15-bp deletion in the second exon of *Asa5G01527.1*, which led to loss of the latter splice site in this exon (Fig. 4e). Accessions harboring mutant and heterozygous alleles displayed significantly late bolting compared with those harboring the wild-type allele (Fig. 4e). These results indicate that *Asa5G01527.1* is a candidate for *qFLT5b*. Five haplotypes (hap1–hap5) were identified in 134 accessions; CG1 and CG2 only possessed a different haplotype (Fig. 4d), indicating that this bolting time-related gene underwent differential selection in two different evolutionary directions. The differential selection of *Asa5G01527.1* may be strongly associated with diversification of bolting time in garlic.

Altogether, our study identified numerous garlic bolting-related candidates that displayed distinct differences in the selective signal across the garlic groups. The differential selection of these bolting-related genes may be responsible for the diversification of bolting time among these groups.

### Selective signatures of cell-specifically-expressed genes related to reproductive transition

**Figure 5:**
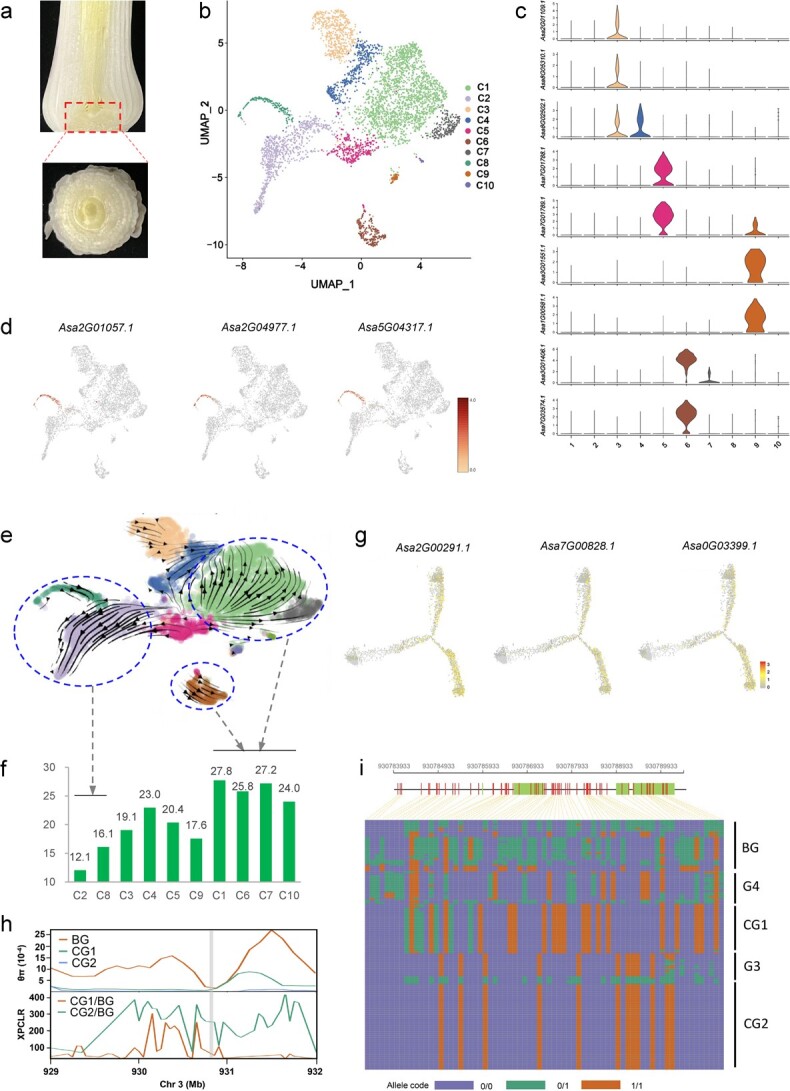
Identification of cell-active genes in garlic basal plate during reproductive transition. **a** Morphological structure of the basal plate in garlic. The longitudinal cut shows the central localization of the apical meristem. The horizontal cut shows numerous leaf bases and the central bud. **b** UMAP visualization of 10 clusters derived from 4491 high-quality cells filtered from the basal plate sample. **c** Violin plots showing the expression of representative marker genes in C3, C4, C5, C6, and C9. **d** Expression pattern of three genes specifically expressed in sieve cells (C8). **e** RNA velocity map of the cells. The direction of the arrows indicates the direction of cell development. **f** Bolting-related active genes in 10 clusters. The ratio was lowest in C2 and C8 cells and highest in C1, C6, C7, and C10 cells. **g** Trajectory along the pseudo-time progression of three bolting-related genes. **h** Distribution of nucleotide diversity (π) and XP-CLR of BG, CG1, and CG2 accessions in the region near *Asa3G03399.1*. **i** Haplotypes for *Asa3G03399.1* in 134 accessions. A distinct loss of genetic diversity in this genetic region was observed in CG1 and CG2.

The garlic inflorescence develops from the apical reproductive meristem, which is located on the basal plate (Fig. 5a). Therefore, genes expressed in basal plate cells may be involved in the transition from vegetative to reproductive development. We explored the genes acting on the basal plate during inflorescence initiation using single-cell RNA sequencing. A total of 5658 cells were identified; 4491 of them were filtered as high-quality cells for further analysis. Cell cluster analysis indicated that these cells could be divided into 10 clusters (Fig. 5b). The number of cells in each cluster ranged from 19 to 1885, and the number of expressed genes ranged from 8229 to 26 347 (Supplementary Data S12). A total of 3066 genes were highly expressed in at least one cell type (Supplementary Data Table S10). Investigation of cluster-specific genes indicated that cells of cluster 3 (C3; ‘cluster’ is abbreviated as ‘C’ hereafter) and C4 were xylem-type, whereas those of C5, C6, C8, and C9 were stem cells, epidermis, sieve, and proliferative cells, respectively (Fig. 5c and d, Supplementary Data S13). These cell identities were further verified by functional enrichment of the cluster-active genes (Supplementary Data S14–S19). Developmental trajectories of basal plate-cells indicated that cells of C5 and C9 developed towards two directions: one was developed as cells of C2 and C8, and another was developed as those of C1, C6, C7, and C10 (Fig. 5e, Supplementary Data S20 and S21).

Among the 3066 genes highly expressed in various cell types (Supplementary Data Table S10), 552 are implicated in garlic bolting by their differential expression at the reproductive stage compared with vegetative growth. A varied ratio of bolting-related active genes was observed across the cell types, and among the clusters this ratio was lowest in C2 and C8 cells, and highest in C1, C6, C7, and C10 cells (Fig. 5f). Notably, C2/C8 and C1/C6/C7/C10 cells are on two different branches developed from the mother cells (Supplementary Data S21), suggesting that cells in the C1/C6/C7/C10 branch are important for garlic bolting. This proposal is supported by two bolting-related genes, *Asa2G00291.1* (Fig. 3c and d) and *Asa7G00828.1* (Supplementary Data S5), that displayed more abundant transcripts in cells on the C1/C6/C7/C10 branch than on the other branch, C2/C8 (Fig. 5g). Among 1417 genes with high expression in C1/C6/C7/C10 cells, 45, 57, 34, and 19 underwent distinct selection in CG1, CG2, G3, and G4, respectively (Supplementary Data Table S11), including the bolting-repressed *Asa2G00291.1* with a selective signature in CG1 and G4 (Fig. 3b).

Interestingly, we identified *Asa3G03399.1*, which encodes a homolog of *Arabidopsis* VERNALIZATION INSENSITIVE 3 (VIN3). Expression of *Asa3G03399.1* can be induced by exposure to a cold environment, but after ~6 days of low-temperature exposure its expression level begins to steadily decrease (Supplementary Data S22). This result revealed that *Asa3G03399.1* exhibits a distinct expression response to vernalization, resulting in similar expression patterns between *Asa3G03399.1* and *Arabidopsis VIN3* [15]. Overexpression of *Asa3G03399.1* altered flowering time in transgenic *Arabidopsis* plants unexposed to low temperatures (Supplementary Data S23). Moreover, the early flowering phenotype of *Asa3G03399.1-*overexpressed *Arabidopsis* was found to be independent of photoperiod conditions (Supplementary Data S23). These findings indicate that *Asa3G03399.1* might have a function similar to that of its homolog *VIN3* in flowering control. We also observed relatively high expression of *Asa3G03399.1* in cells on the C1/C6/C7/C10 branch (Fig. 5g). In aggregate, our evidence suggests that basal plate cell-active *Asa3G03399.1* is involved in reproductive transition via the vernalization response. Notably, *Asa3G03399.1* underwent distinct selection in both CG1 and CG2 (Fig. 5h), causing a loss of diversity in these two cultivated groups (Fig. 5i). Selection of *Asa3G03399.1* may be responsible for the evolution of bolting ability in cultivated accessions of CG1 and CG2.

### Selection of genes potentially associated with abnormal flower growth

**Figure 6:**
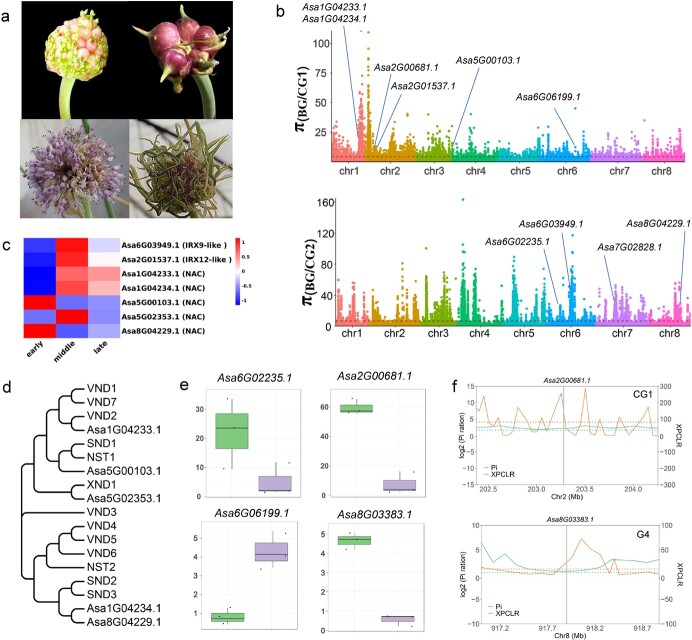
Selection signatures in genes related to flower differentiation in garlic. **a** Variants of the morphological structure of garlic inflorescence: numerous flowers and bulbils in the same inflorescence (upper left); large bulbils, flowers aborted (upper right); inflorescence contains mostly flowers (lower left); flowers are aborted, thin leaves and bulbil dominant in the inflorescence (lower right). **b** Selection indicators of two cultivated CG1 and CG2 accessions from the top 5% of π_BG_/π_CG_. Candidate genes identified in this study are marked. **c** Expression level of candidate genes associated with flower development at different developmental stages of the inflorescence in F87. **d** Phylogenetic tree of differentially expressed NAC proteins of garlic and *Arabidopsis* cell wall-regulated NAC proteins. **e** Box plots show FPKM values of four candidate genes in the flower of bulbil-producing cv. ‘Ershuizao’ (left) and bulbil-free F87 (right). **f** Distribution of XP-CLR and the ratio of nucleotide diversity in the region near *Asa2G00681.1* and *AsaG03383.1*.

Abortion of anthers and stigmas and formation of inflorescence bulblets are two of the main causes of abnormal flowers. Among the investigated accessions of BG, 20 accessions bolted, and 14 of these produce normal flowers. All accessions from the other four groups either failed to bolt or produced degenerated flowers with withered anthers and stigmas, and numerous inflorescence bulblets/topsets (Fig. 6a). To identify genes associated with flower development in garlic, we compared the expression profiles of flowers at three different developmental stages (early, middle, and late) from the fertile accession F87 (transcriptome data reported by Shemesh-Mayer *et al*. [16]), and identified 2531 and 2066 genes with expression changes from the early stage to the middle stage of flower growth and from the middle to late stage of flower differentiation, respectively (Supplementary Data S24; Supplementary Data Tables S12 and S13), indicating that these genes are potentially involved in flower development. Selection signals of flower development-related genes were detected in all groups except G3, because most G3 accessions did not bolt and produce flowers. We found that 134, 123, and 70 flower genesis-related genes underwent distinct selection in CG1, CG2, and G4, respectively, including 18 genes with significant selective signals in two of the garlic groups (Supplementary Data S25). Notably, of these DEGs related to flower development, many are homologous to known cell wall biosynthesis genes, and have undergone distinct selection during garlic evolution history, including *IRX12*-like *Asa2G01537.1* (CG1) and *IRX9*-like *Asa6G03949.1* (CG2; Fig. 6b and c, Supplementary Data S26). In addition, 21 garlic NAC genes displayed expression differences between different stages of flower development (Supplementary Data S27), and five of these encoded orthologs of the *Arabidopsis* cell wall formation NAC proteins (Fig. 6d), with distinct selection signatures in CG1 (*Asa1G04233.1*, *Asa1G04234.1*, and *Asa5G00103.1*), CG2 (*Asa8G04229.1*), and G4 (*Asa5G02353.1*) (Supplementary Data S26). Selection of these cell wall formation-regulated genes is potentially responsible for abnormal growth in the anthers and stigmas of flowers in cultivated garlic.

The presence of an inflorescence bulblet is assumed to be another factor that causes abnormal flowers in garlic [26, 27]. Morphologically, bulblets are vegetative organs similar to cloves that grow from underground on the leaf bases. Some genes related to clove enlargement displayed distinct differences in expression between bulblet-producing and bulblet-free accessions. For example, *Asa2G00681.1* and *Asa6G02235.1*, two bulb swelling-promoting genes [12], showed higher expression levels in the flowers of bulblet-producing cv. ‘Ershuizao’ (transcriptome data of flower reported by Sun *et al*. [13]) in comparison with bulblet-free F87, whereas *Asa6G06199.1*, which is involved in repressing bulb swelling [12], is downregulated in ‘Ershuizao’ compared with F87 (*P* < .05; Fig. 6e). Notably, these three genes underwent distinct selection in CG1 or CG2 (Fig. 6f; Supplementary Data S9). Additionally, we identified the GA2ox-encoding *Asa8G03383.1*, whose expression was significantly different between the flowers of bulblet-producing ‘Ershuizao’ and bulblet-free F87 plants (*P* < .05; Fig. 6e), indicating that this gene potentially plays a role in formation and swelling of the inflorescence bulblet. Importantly, we found that *Asa8G03383.1* was subject to selection in G4 (Fig. 6f). The selection of these genes may be associated with the evolution of bulblet organogenesis in the flowers of cultivated garlic.

## Discussion

Wild crop relatives and landraces generally possess greater genetic diversity than cultivated crops and can provide valuable resources for breeding and crop improvement [17]. In garlic, primitive landraces from Central Asia flower and produce fertile seeds, but the genetic resources underlying these abilities are lost in cultivated varieties [7]. A recent study sequenced 84 accessions, reported 129.4 million variations, and analyzed the evolution of bulb development in garlic [12]. However, previous analyses did not include the flowering germplasm; therefore, genomic characterization of reproductive traits was not performed. In the present study, 50 new garlic accessions, including 14 flowering genotypes, were analyzed for genomic variation. Because of the greater diversity of these resources, whole-genome sequencing yielded twice as much genomic variation as seen in the previously reported 84 accessions [12]. Five genetic groups found in this study correspond to a previously proposed biogeographical and morphological classification of the garlic crop [14], while the previous report based on 84 accessions identified only four genetic groups [12]. Based on genomic analysis, we propose that garlic domestication evolved in two independent directions, which further supports the previous finding about independent domestication of Chinese cultivated CG1 and CG2 [12].

According to the *F*_ST_ values, the predicted divergence between CG1 and CG2 should be larger than that between G3 and G4. However, this study found a larger overlap in the selective sweeps between CG1 and CG2 than between G3 and G4. CG1 and CG2 are widely cultivated groups that possess good bulb traits and are probably selected mainly for bulb production [12]. During the evolution and domestication of CG1 and CG2, the genomic regions associated with their agronomic traits underwent common selection. Taking bolting ability as an example, five genes associated with the transition from the vegetative to the reproductive stage had significant selective signals in both CG1 and CG2, including the bolting ability-associated *Asa5G01527.1* and *Asa3G03399.1*, whereas only two of these genes were identified in G3 and G4. The common selection of traits should be an important reason for the greater overlap of selective sweeps between CG1 and CG2, although their genomes possess large divergence. Therefore, this study provides novel insights into garlic evolution.

Garlic bolting is determined by the transition from vegetative growth to reproductive growth. Approximately 47.1 and 87.5% of the accessions in G3 and G4, respectively, failed to bolt, indicating a distinct degeneration in bolting ability. Interestingly, we found a remarkable recovery in the ratio of bolting accessions in cultivated CG1 and CG2, which might have resulted from the consumption of flower stems in the cultivation areas, resulting downstream in selection for bolting traits. In model plants, vernalization is an important environmental factor that induces flowering transition, and *VERNALIZATION INSENSITIVE 3* (*VIN3*) plays an important role in regulating this process [18]. Temperature and photoperiod are other environmental factors that determine flowering, and the CO-FT module plays a central role in this process. CO-FT module function is highly conserved in plants, including garlic [19, 20], which can be mediated by *BBX* genes, such as *Arabidopsis BBX19*, which can interact with *CO* to repress *FT* transcription [21]. In this study, hundreds of genes related to garlic bolting were found to have significant selection signals during garlic evolution and domestication, including *BBX*-encoding *Asa2G00291.1* and *VIN3*-like *Asa3G03399.1*. Additionally, GTE proteins are essential for mediating the abscisic acid (ABA) response by interacting with the BTB domain-containing BT2 protein [22], and ABA plays a crucial role in controlling the floral transition of *Arabidopsis* [23]. This study identified a GTE encoding *Asa5G01527.1* involved in garlic bolting that has a distinct selective signature. The selection of these genes should have been responsible during the evolution of garlic bolting.

The abnormal development of flowers, such as abortion of anthers and stigmas, is another challenge in the reproduction of cultivated garlic. Cell wall composition and function in the anther and stigma are pivotal for proper flower differentiation. The cell wall mainly consists of cellulose, lignin, pectin, and hemicellulose, and large numbers of enzymes are involved in the biosynthesis of these components. Therefore, loss of function of these enzyme-encoding genes frequently causes abnormal flowers [24]. NAC proteins have been identified as master switches that trigger the biosynthesis of cell wall components [25]. In this study, the distinct selection of numerous cell wall formation-related genes potentially associated with flower development might play a role in the degeneration of anthers and stigmas in cultivated garlic flowers.

Bulblets have been identified as an important factor in the abnormal development of flowers because their expanded growth is deemed to plunder nutrients essential for flower bud growth. Previous studies have proposed that continuous removal of bulblets during inflorescence development could promote flower development [26, 27]. Morphologically, bulblets are vegetative organs. Gibberellins (GAs) are crucial hormones that inhibit swelling of vegetative organs such as potato tubers and garlic bulbs [28, 29]. GA2ox is a pivotal enzyme that catalyzes the hydrolysis of bioactive GAs [30]. Interestingly, this study identified several bulb-swelling growth-related genes that showed differing expression between bulblet-free and bulblet-producing garlic flowers, including GA2ox-encoding *Asa8G03383.1*, which showed distinct selective signatures. Their selection is logically associated with the evolution of bulblets in cultivated garlic.

Altogether, flowers have become non-essential for garlic reproduction, causing reproductive degeneration such as low bolting ability, abortion of anthers and stigmas, and the presence of bulblets. This study reveals that numerous genes related to these traits show distinct selection signatures, and that their selection should be an important force driving the degeneration of corresponding reproductive traits. As garlic relies on vegetative propagation, some disadvantageous alleles of reproductive genes should experience relaxed selection. Although it is difficult to explore relaxation of selective pressure on reproductive genes based on evidence from this study, our results provide important insights into the evolution of reproductive traits in garlic.

## Materials and methods

### Plant material and phenotypic assessment

Forty-one garlic accessions were grown in an experimental plot in Northern Israel. In 2020, the young leaves were collected, dried, and sent to Shanghai OE Biotech. Co. Ltd (Shanghai, China) for DNA extraction. In 2018, nine additional accessions were grown in Jinxiang, Shandong Province, China. In 2021, a new pool of 50 DNA samples was obtained. A combined analysis of these 50 accessions together with the 84 accessions reported by Li *et al*. [12] was performed (Supplementary Data Table S4). A population consisting of 230 garlic accessions for a genome-wide association study (GWAS) of bolting time was grown on the experimental farm of the Shandong Dongyun Research Center of Garlic Engineering, Jinxiang, China, from 2018 to 2019. The date of the first appearance of the flower stalk was recorded for 30 individuals of each accession. The number of days from planting to bolting was used to quantify the bolting time.

### Genome sequencing

Genome sequencing was performed at Shanghai OE Biotech. Co. Ltd (Shanghai, China). Extraction of the genomic DNA (gDNA) of each accession was carried out using fresh leaves. After determining the quality and quantifying the concentration, gDNA sequencing libraries were developed with the TruSeq Nano Sample Prep Kit (Illumina Inc., San Diego, CA, USA). In brief, ~1.5 μg of gDNA was sonicated into fragments ~350 bp in size, and the resulting DNA fragments were then processed and ligated to full-length adapters. Then, after PCR amplification, the corresponding PCR-amplified products were purified via the AMPure XP Bead system. Subsequently, the size distribution of the generated libraries was analyzed via the Agilent 2100 Bioanalyzer. Finally, each library was sequenced on the Illumina HiSeq X platform to generate 150-bp reads.

### Variation calling

Prior to calling the variation, reads from Illumina sequencing were filtered by default parameters using the FastQC software package (version 0.11.9; https://www.bioinformatics.babraham.ac.uk/projects/fastqc/) to remove base calling duplicates and adapter contamination due to the generated low-quality paired reads. Subsequently, mapping of the generated high-quality reads to the garlic reference genome (GCA_014155895.2) was executed via BWA software (version 0.7.8) [31], and the results of alignment were converted to BAM format via SAMtools (version 1.3) [32]. Thereafter, variations from the genome of each accession were explored based on the Bayesian approach implemented in the SAMtools package and filtered using the following criteria: genotype quality of each individual <5, depth of each individual <3, and QUAL <20. The ANNOVAR package [33] was used to annotate the identified SNPs based on the gene annotation and to categorize the SNPs according to their positions, including intergenic, intronic, exonic, splicing sites, and 1-kb downstream and upstream regions. In addition, functional effects of each variant, including non-synonymous, synonymous, non-frameshift, frameshift, and stop loss and gain, were determined via the ANNOVAR package [33].

### Inference of genetic diversity, population structure, and demographic history

The program TreeBest (version 1.92) [34] was used to explore the phylogenetic tree of the investigated accessions based on the maximum likelihood (ML) method by *p*-distance, with 1000 bootstrap iterations. The GCTA package [35] was used to analyze the PCA of these studied accessions, and the significance levels were tested via the Tracy–Widom test. The ADMIXTURE program (version 1.3.0) [36] was used to investigate the population structure, and each *K* value was run 10 times using different random seeds. The *Q*-matrices were aligned using pong software (version 1.4.7) [37] and were clustered according to similarity. Subsequently, the matrices from the largest cluster were used to produce the final matrix of admixture proportions. To estimate the genetic diversity, after removing windows with fewer than five SNPs, the nucleotide diversity (θπ) [38] within each group and the *F*_ST_ between two groups [39] were calculated using the VCFtools package (version 0.1.14) [40], using genome-wide SNPs, based on 500-kb sliding windows. The demographic histories of the five garlic groups were inferred via the PSMC model [41]. To convert scaled times and population sizes to actual times and sizes, a generation time of one year and a rate of 2.5 × 10^−9^ mutations per nucleotide per year were used [42]. Due to the limited number of recombination events that have occurred over a short period of time, it is difficult to estimate the recent population size with the PSMC method [41], and therefore we also inferred the population history of garlic with a coalescent simulation-based composite likelihood method using quadruple degenerate sites through the fastsimcoal package (version 2.5.1) [43].

### Selective sweep scanning

Three statistical methods were used to identify genome-wide selection signals using the genome-wide SNP sets, i.e. the PBS approach, the test of cross-population composite likelihood ratio (XP-CLR; https://github.com/hardingnj/xpclr) [44], and the nucleotide diversity (θπ) comparison. Of these, the PBS method could detect some incomplete selective sweeps resulting from short divergence times based on the comparison of pairwise *F*_ST_ between designed compared patterns within the corresponding sliding window (500-kb windows sliding in 125-kb steps) [45]. The XP-CLR method was calculated with the following parameters: sliding window size 0.01 cM, grid size 50 k, maximum number of SNPs within a window 100, and correlation value for two SNPs 0.95. Windows with the top 5% of XP-CLR scores were deemed to be outliers. For the θπ comparison, θπ was estimated in A and B populations using a sliding window as above, and the statistic log_2_(θπ_A_/θπ_B_) was then calculated with regard to the populations A and B, resulting in a top positive value (5% outliers) that represents a putative selection in population B. Notably, the method of θπ comparison is a classic statistic for detecting selective signals (especially hard sweeps) through the assumption that selected regions have a decrease in genetic diversity, and the PBS method is powerful for detecting incomplete selective sweeps [46], whereas the XP-CLR method has power to find the ancient selective signals [44]. Therefore, the combination of these three methods is suitable for detecting different selective events. Selective signals from at least two of these three statistical methods were deemed to be putative selective sweeps.

### Expression analysis

Transcriptome analysis for identifying genes associated with the flowering transition was performed using the garlic variety ‘Yuanjiangyangsuan’. Briefly, in October 2019, cloves were planted at the farm of the Institute of Bast Fiber Crops, Chinese Academy of Agricultural Sciences, Changsha, China. Fresh leaves were collected in February and March 2020, when the garlic plants were in the vegetative and reproductive stages, respectively. Three individual plants were used as replicates in both vegetative and reproductive stages. Total RNA extracted from the collected leaves was used to construct complementary DNA (cDNA) libraries with a fragment length of 300 bp via a NEBNext^®^ Ultra™ RNA Library Prep Kit for Illumina^®^ (New England BioLabs, Ipswich, MA, USA). Each library was subjected to Illumina paired-end sequencing using the HiSeq X platform. After trimming the adapter sequences and filtering low-quality reads, the generated clean reads were used to identify DEGs between vegetative and reproductive garlic plants. Additionally, Illumina reads from the RNA sequencing of flowers at three different developmental stages (early, middle, and late) from fertile garlic accession F87 were downloaded from the NCBI Sequence Read Archive (SRA) database (bioproject no. PRJNA264944) and were used to identify DEGs during the flower development stages. To quantify the transcript abundance of garlic genes, clean reads from each sample were compared with the garlic reference (GenBank accession number GCA_014155895.2) by default parameters using hisat2 software (version 2.2.1.0) [47]. Fragments per kilobase per million reads (FPKM) [48] were estimated and were used to quantify the expression level of each gene. DEGs between the leaves at different developmental stages were determined using the DEseq program (version 1.18.0) [49], and expression with >2-fold difference was deemed significant (*P* < .05).

### Genome-wide association study

Based on the efficient mixed model association acceleration program [50], GWAS analysis was performed for germination time traits using GBS SNPs (MAF ≥0.01, deletion rate ≤0.5) reported by Li *et al*. [12] for 230 garlic cultivars. Population stratification and hidden relatedness were estimated using a kinship (K) matrix generated from the FaST-LMM program [51]. The *P*-value threshold for suggestive association loci was set to 1.0 × 10^−6^, which was estimated based on Bonferroni correction for the effective number of independent markers [52].

### Real-time PCR and overexpression of *Asa3G03399.1*

The bolting variety of garlic, Cangshansuan, was used for the real-time PCR (qRT–PCR) of specific genes. Cloves were planted in pots and grown at 22°C. After 2 weeks, plants were exposed to 4°C. The garlic bulb was collected after the plant had been exposed to a cold environment for 0, 3, 6, 9, 12,15, and 18 days. Plants at different developmental stages were selected for sampling and rapidly placed in liquid nitrogen and stored at −80°C. RNA was extracted from garlic plants and converted to cDNA, and *Asa3G03399.1* was quantified by qRT–PCR using the *Cyclophilin* (*CYP*) gene as an internal control. Supplementary Data Table S14 lists the primer sequences used in this experiment. The relative expression levels of the genes were determined with reference to a method described previously [53]. Full-length cDNA of *Asa3G03399.1* from high-fidelity amplification was ligated to a PBI121 vector and its expression was driven by a CaMV 35S promoter. The resulting vector was used to transform *Arabidopsis* based on the method of floral dipping [54].

### Statistical and phylogenetic analyses of candidate proteins

Statistical analyses, such as the correlation analysis between the abundance of gene transcript and bolting time in the 81 garlic accessions (transcriptome data reported previously [12]), were carried out using SPSS software. Phylogenetic analysis of proteins was carried out in a two-step process: sequence alignment was performed using the Clustal program [55], followed by construction of an unrooted phylogenetic tree by the neighbor-joining (NJ) method using MEGA software and bootstrap testing with 1000 replicates [56].

### scRNA sequencing and cell clustering

The bolting cultivar ‘Ershuizao’ was used for scRNA. In October 2021, cloves were planted on a farm at the Institute of Bast Fiber Crops, Chinese Academy of Agricultural Sciences, Changsha, China. On 10 January 2022, when the inflorescence buds were observed, the basal plates of the bulbs were collected to extract the protoplasts. After testing protoplast viability based on trypan blue staining, the protoplasts were used to construct a library via a Chromium Next GEM Single Cell 3 GEM Library and Gel Bead Kit version 3.1 (10× Genomics, USA). Then, the generated library was quantified by a high-sensitivity DNA microarray on an Agilent 2100 system (Agilent, USA) and Qubit high-sensitivity DNA analysis (Thermo Fisher Scientific, USA). Finally, paired-end sequencing of these libraries was performed using an Illumina NovaSeq 6000 system (Illumina, San Diego, USA). The 10× Genomics software Cell Ranger (version 5.0.0; https://support.10xgenomics.com/single-cell-gene-expression/software/pipelines/latest/what-is-cell-ranger) was used to perform quality statistics and to align the scRNA-seq read with the reference genome. Based on the initial quality control of Cell Ranger, the data were further used to perform quality control with the Seurat software package (version 4.0.0) [57]. Low-quality cells were screened based on the distribution of indicators (e.g. nUMI, nGene, and percent.mito, and double cells), and were removed using DoubletFinder software (version 2.0.2) [58]. Highly variable genes (HVGs) were filtered using the FindVariableGenes function in the Seurat software package, and were used for principal component dimensionality reduction analysis, based on their expression in cells. After removing the batch effect using the Batchelor software package via the mutual nearest neighbor (MNN) method [59], the cells were grouped based on expression profiles of genes in each cell, via the FindClusters function in the Seurat package. Visualization of the results was performed using a 2D UMAP algorithm. Marker genes were identified via the FindAllMarkers function in the Seurat package, and were visualized using the VlnPlot and FeaturePlot functions.

### Assessment of cell identity, pseudo-time, and RNA velocity analysis

Cell identity of each cluster was inferred based on two methods. Briefly, the cell-specifically-expressed genes were identified as marker genes, and their annotations were used to infer the type of the corresponding cells. Then, GO and KEGG analyses of high-expressed genes in each cell cluster were further used to infer the cell type. The dynamics of the pseudo-time for cell development were simulated by the Monocle2 package (version 2.9.0), based on the expression patterns of marker genes [60]. In brief, raw counts were converted from Seurat objects to CellDataSet objects using the Import CDS function in Monocle. Then, the differentialGeneTest function of the Monocle2 package was used to select sorting genes (qval <0.01) that may be informative regarding sorting cells along pseudo-time trajectories. Subsequently, a reduced-dimensional clustering analysis was performed via the reduceDimension function, and trajectory inference was performed using the OrderCells function. The Python script velocyto.py (https://github.com/velocyto-team/velocyto.py) was used to perform RNA velocity analysis, recalculating spliced and unspliced reads using the Cell Ranger output folder [61]. RNA velocities (transcription, splicing, and degradation rates) for single cells were calculated by constructing the likelihood-based dynamic model and velocity graph using scVelo (https://scvelo.readthedocs.io/ [62]).

## Supplementary Material

Supplementary_Figure_uhad208

Supplementary_Tables_uhad208

## Data Availability

The reported variation data from whole-genome sequencing have been deposited in the Genome Variation Map (GVM) in the Big Data Center, Beijing Institute of Genomics (BIG), Chinese Academy of Science, under the project accession numbers PRJCA006629 and PRJCA012274. The sequence reads of single-cell RNA sequencing have been deposited in the Genome Sequence Archive in the National Genomics Data Center, China National Center for Bioinformation/Beijing Institute of Genomics, Chinese Academy of Sciences (GSA: CRA009173).
