# Supplementary material for: Genome resequencing reveals the evolutionary history of garlic reproduction traits
Source: Hortic Res. 2023 Oct 17;10(11):uhad208. doi: 10.1093/hr/uhad208 (PMC10689055; doi:10.1093/hr/uhad208)
Supplement: Supplementary_Figure_uhad208 [file supplementary_figure_uhad208.docx]

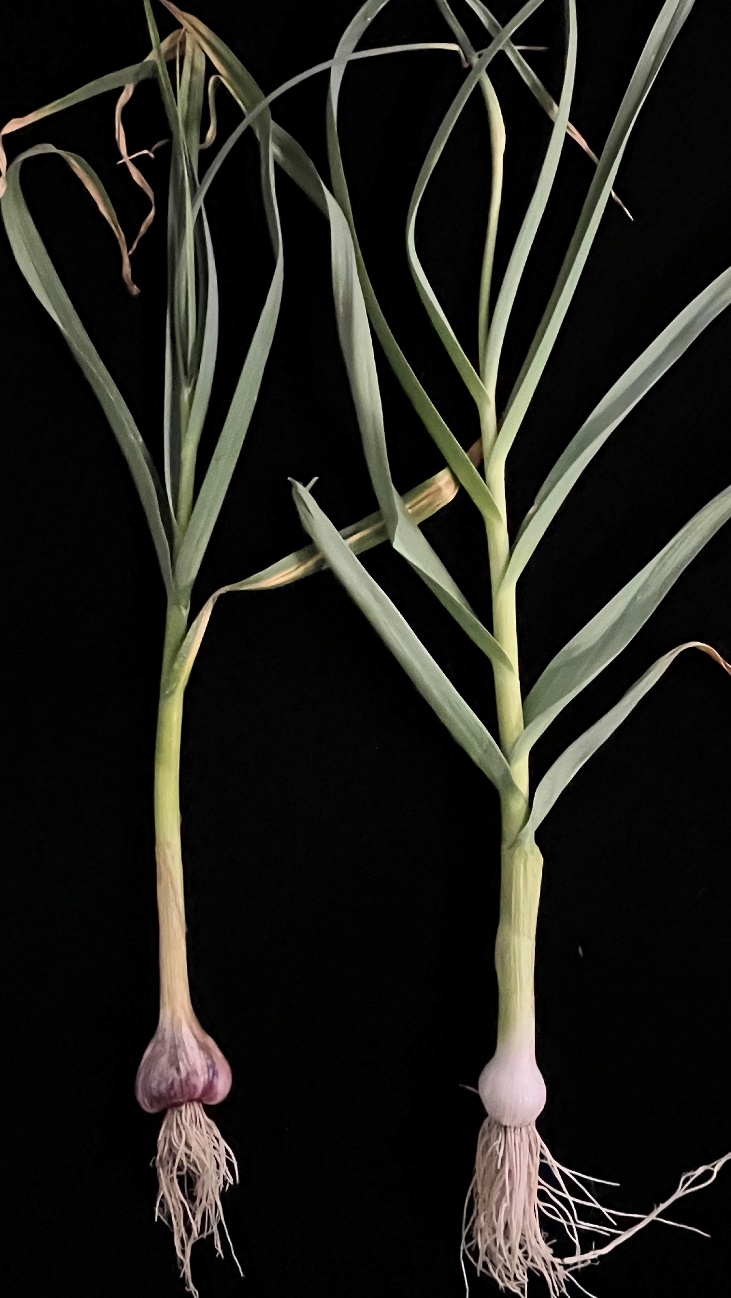


**Fig S1** Whole plant of garlic from two accessions. Garlic in left fails in bolting.


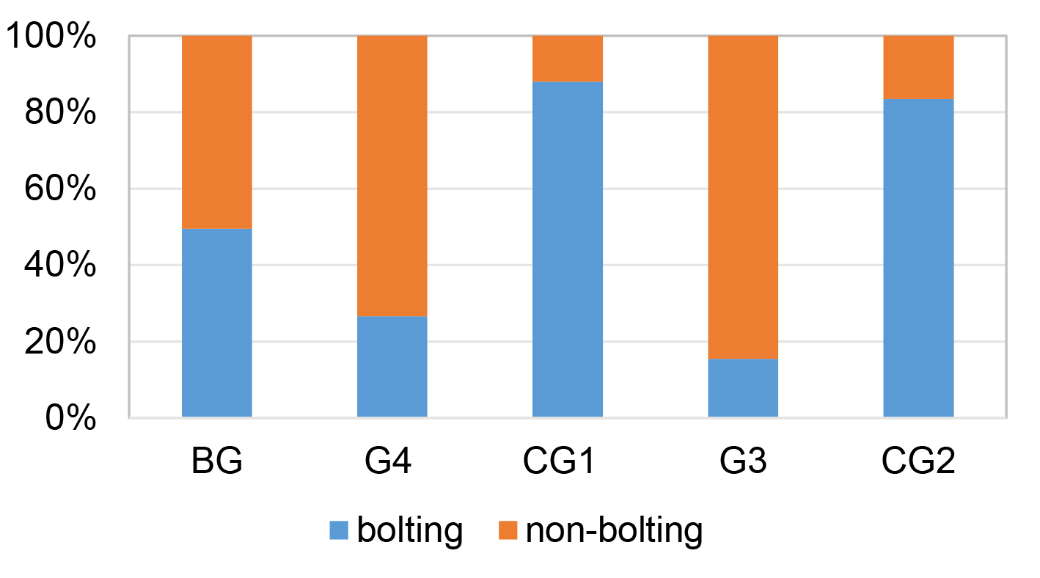


**Fig S2** Ratio of bolting and non-bolting accessions in five garlic groups.


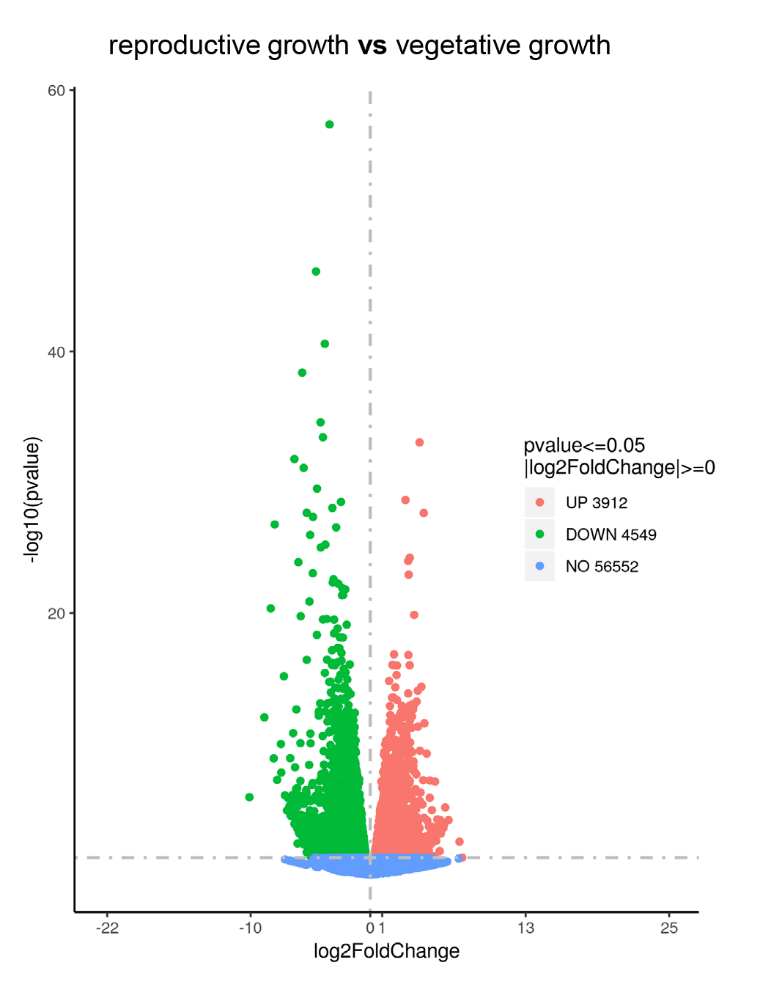


**Fig S3** Differentially expressed genes in the leaves of garlic under vegetative and reproductive growth in the variety Yuanjiangyangsuan. Red dots represent genes with more transcripts in the library of reproductive growing garlic, green dots represent genes with fewer transcripts in the library of reproductive growing garlic, and grey dots indicate genes whose expression are not changed significantly.


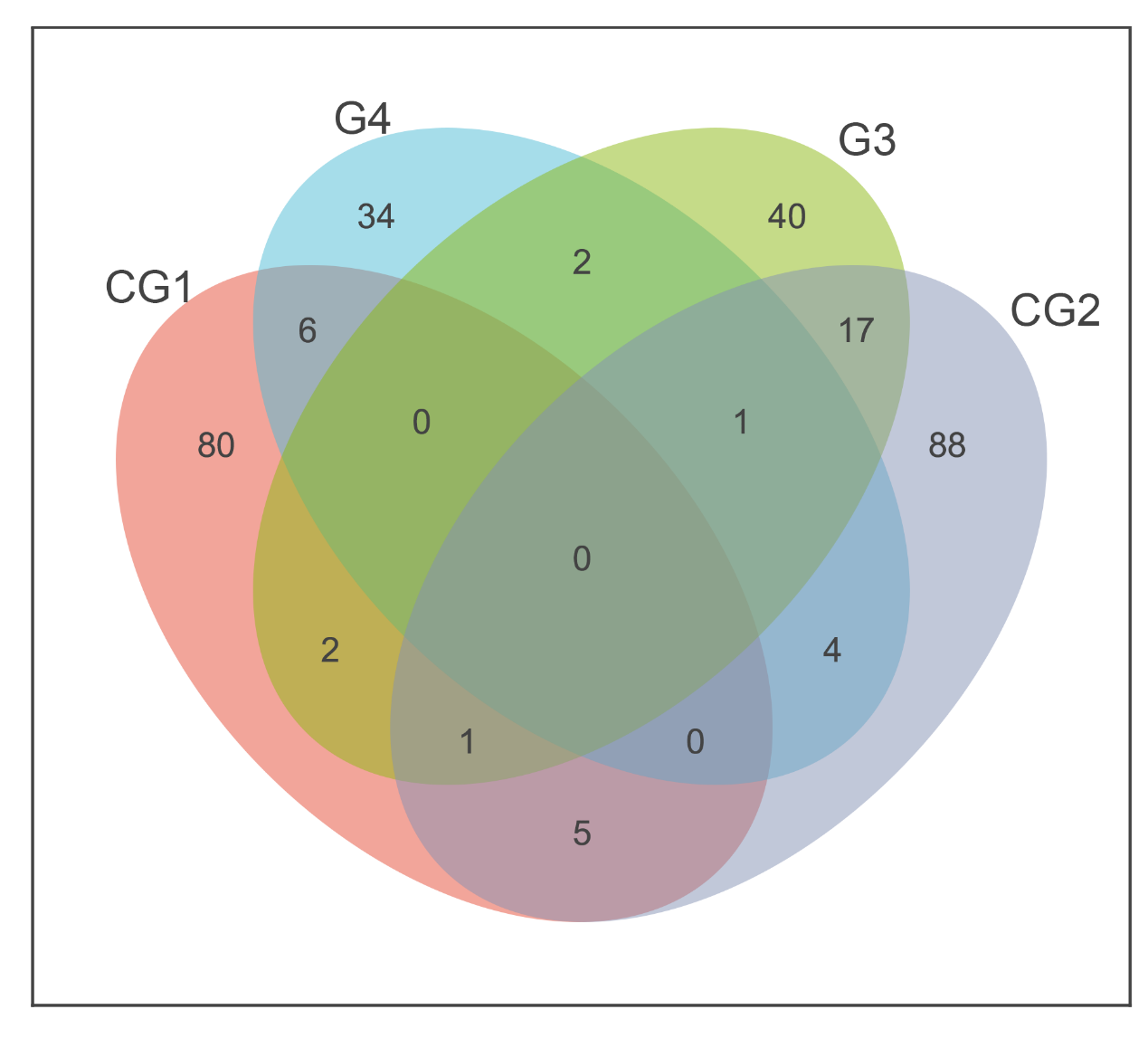


**Fig S4** Venn diagram of differentially expressed genes with selective signal in CG1, G4, G3, and CG2, respectively.


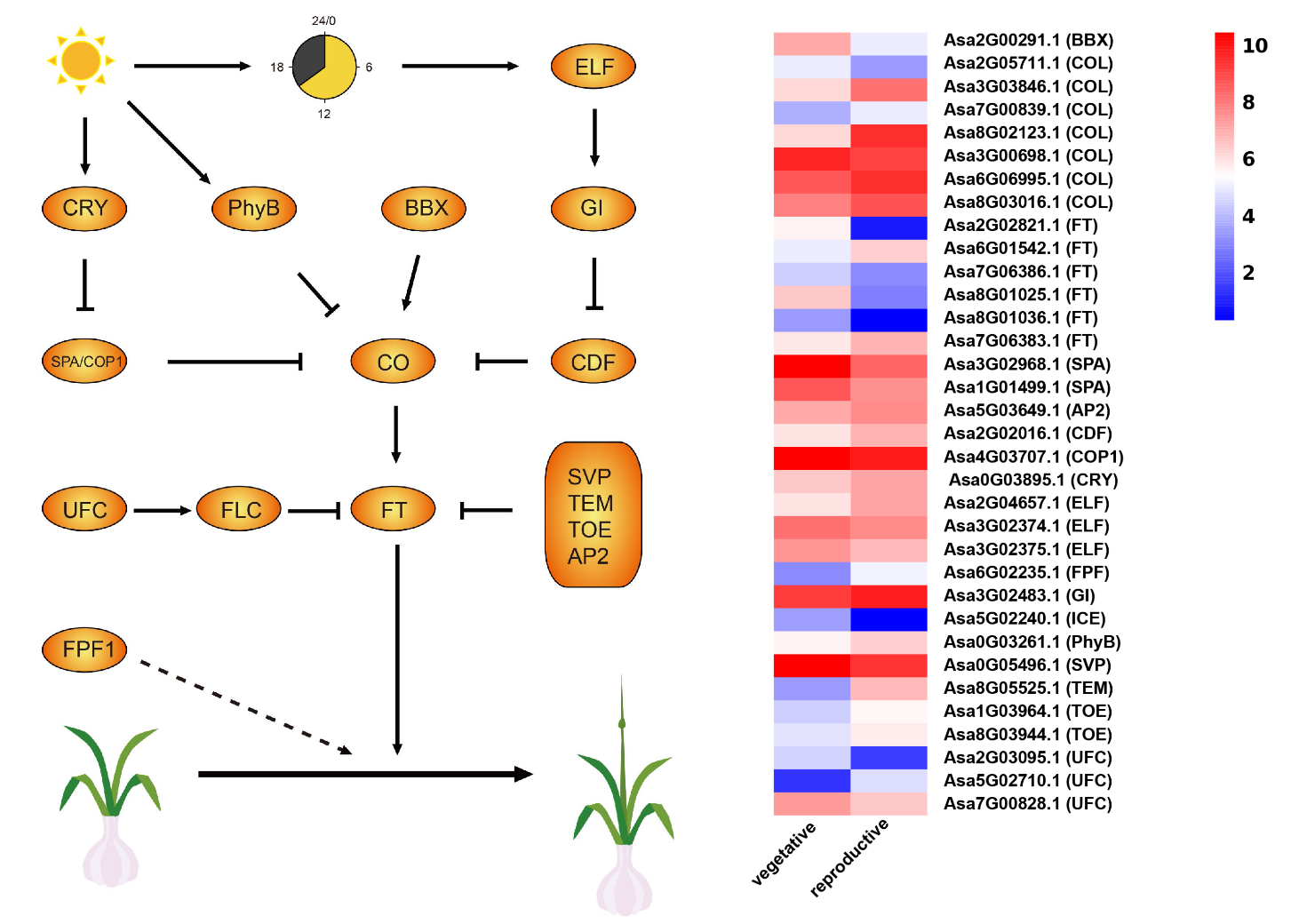


**Fig S5** Putative regulatory network for controlling garlic bolting, which consist of 34 differentially expressed genes that encode the homologs of Arabidopsis flowering-controlled proteins.


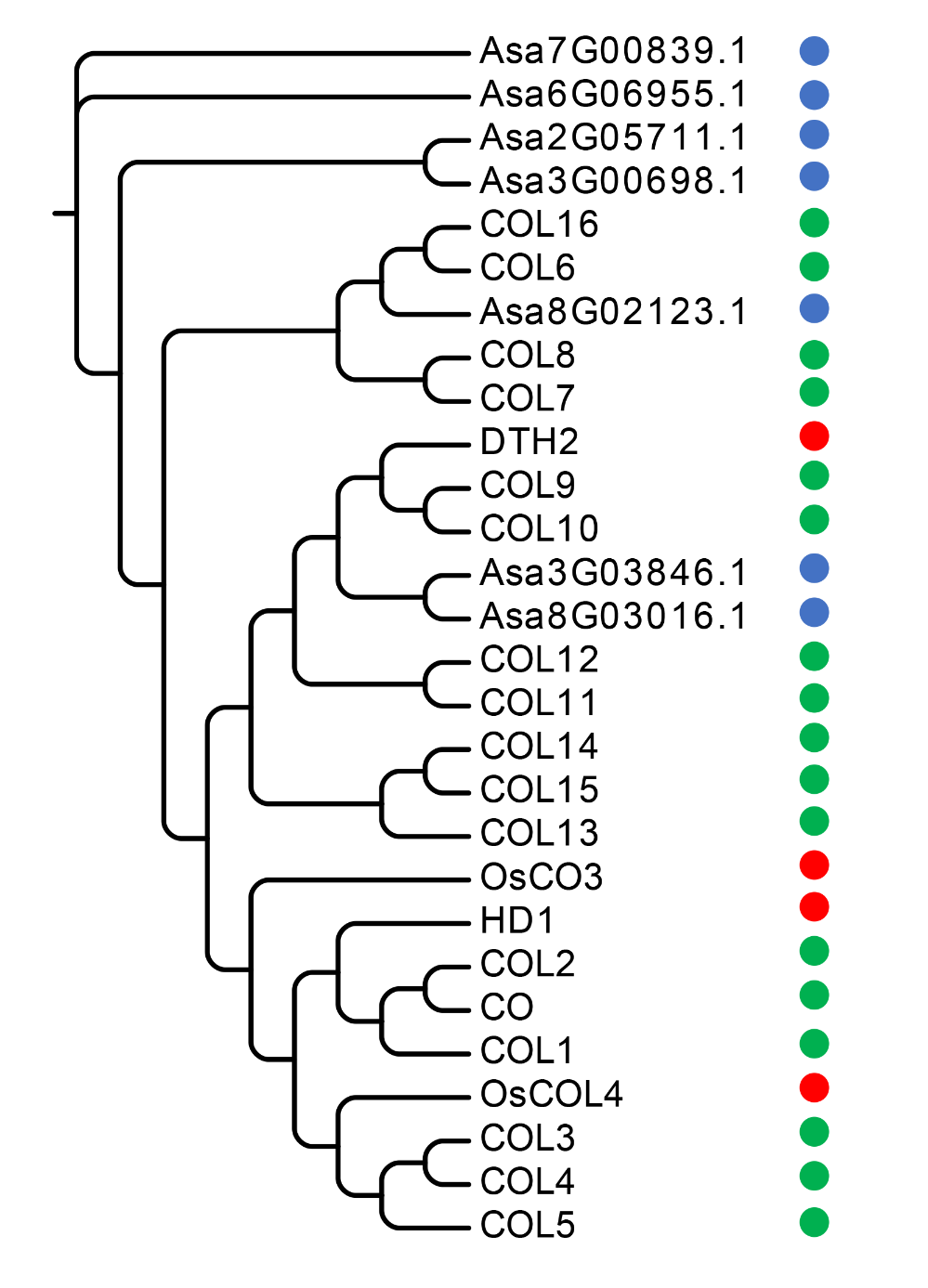


**Fig S6** Phylogenetic tree of differentially expressed CO-like genes of garlic (blue) and known flowering time-controlled CO/CO-like genes of rice (red) and Arabidopsis (green).


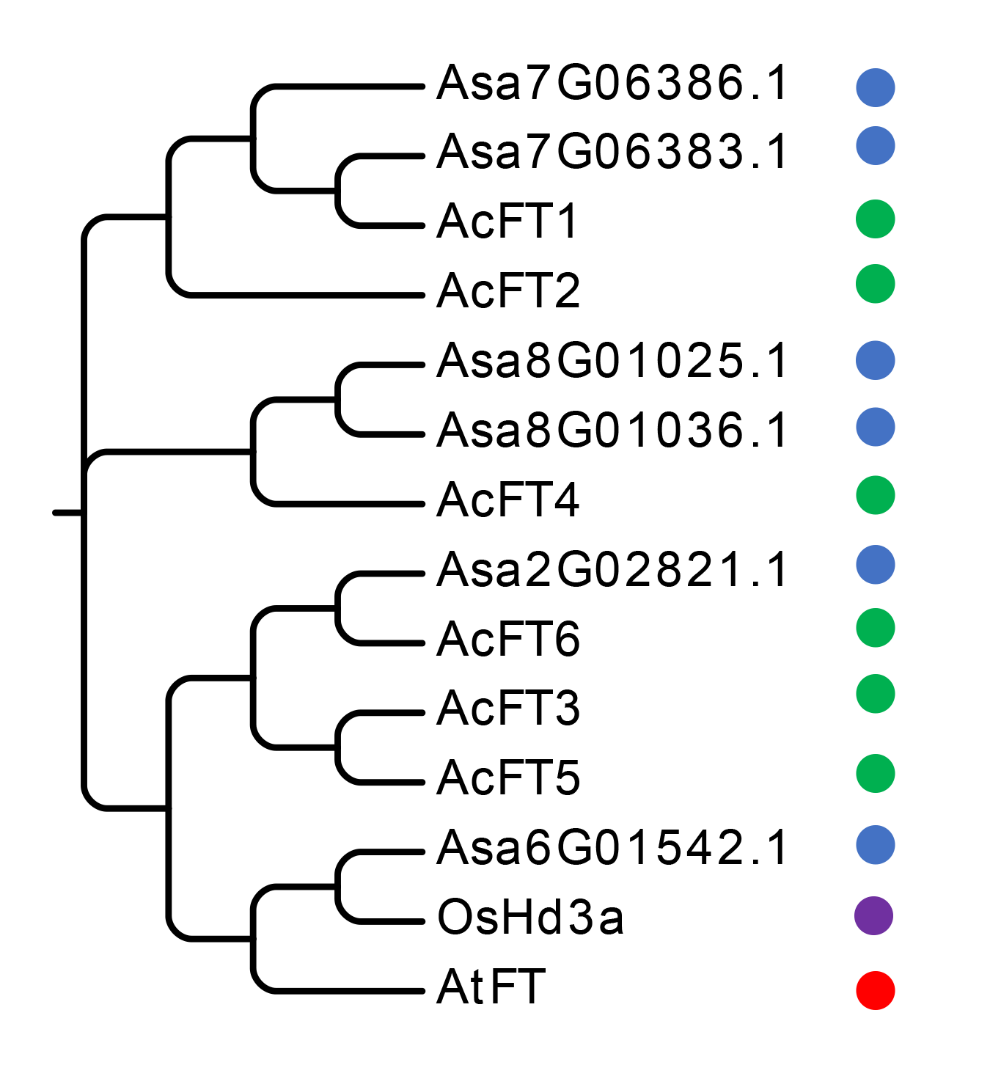


**Fig S7** Phylogenetic tree of differentially expressed FT-like genes of garlic (blue) and known flowering time-controlled FT/FT-like genes of rice (purple), Arabidopsis (red), and onion (green).


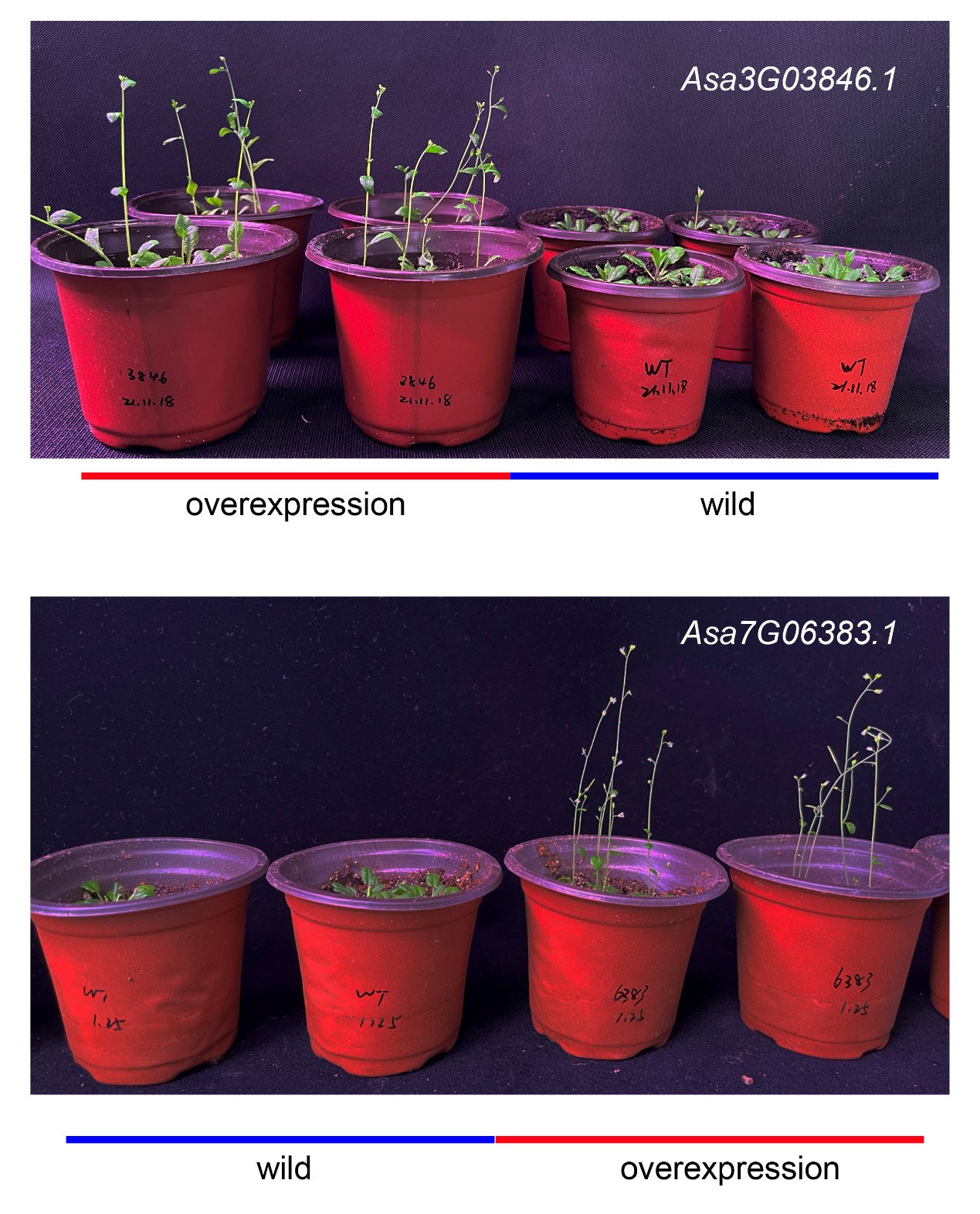


**Fig S8** Comparison of the flowering time between wild and overexpressed Arabidopsis for two genes, *Asa3G03846.1* and *Asa7G06383.1*.


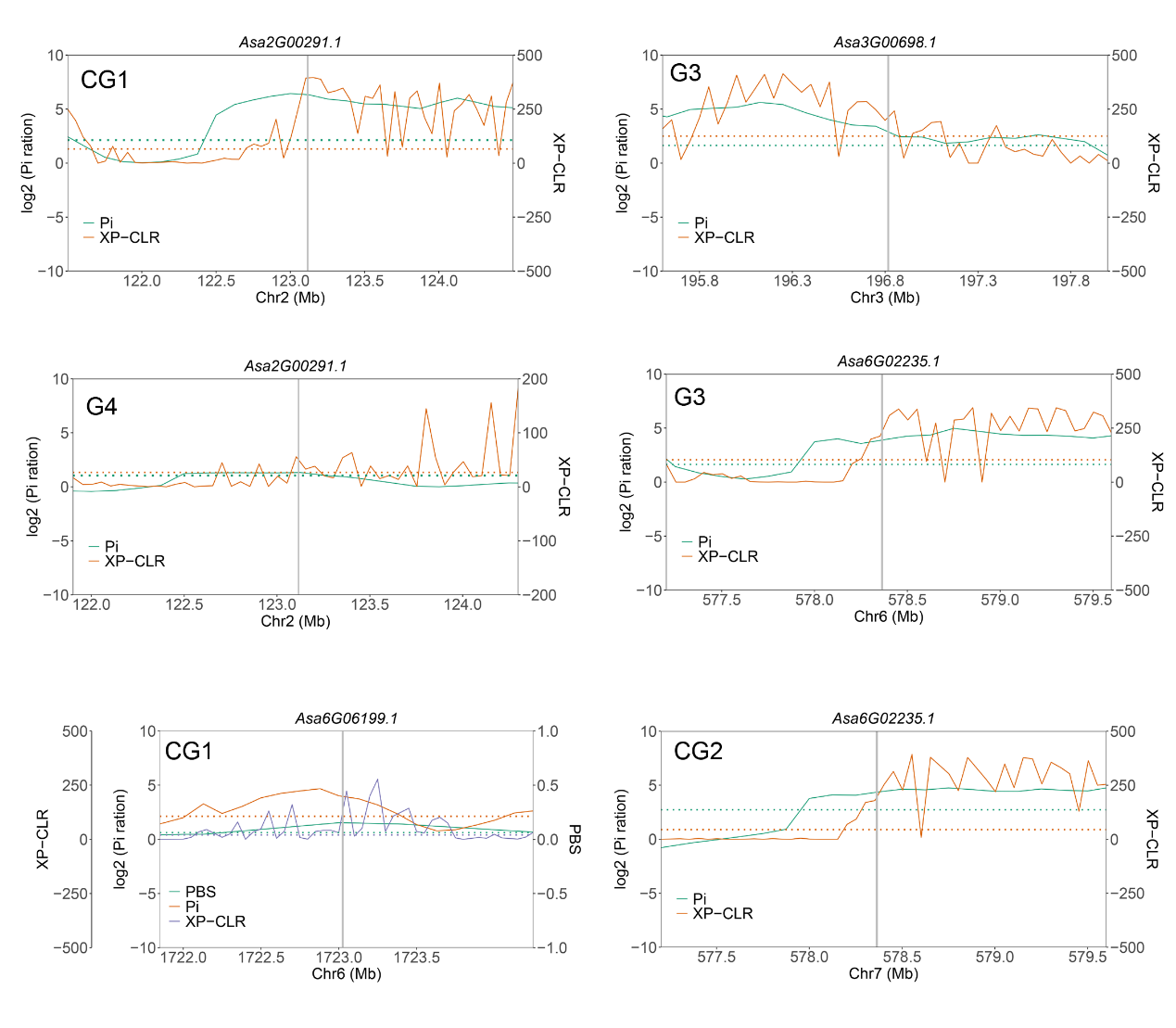


**Fig S9** Distribution of the ratio of nucleotide diversity, XP-CLR and *F_ST_* values in the region near to the bolting time-related candidates.


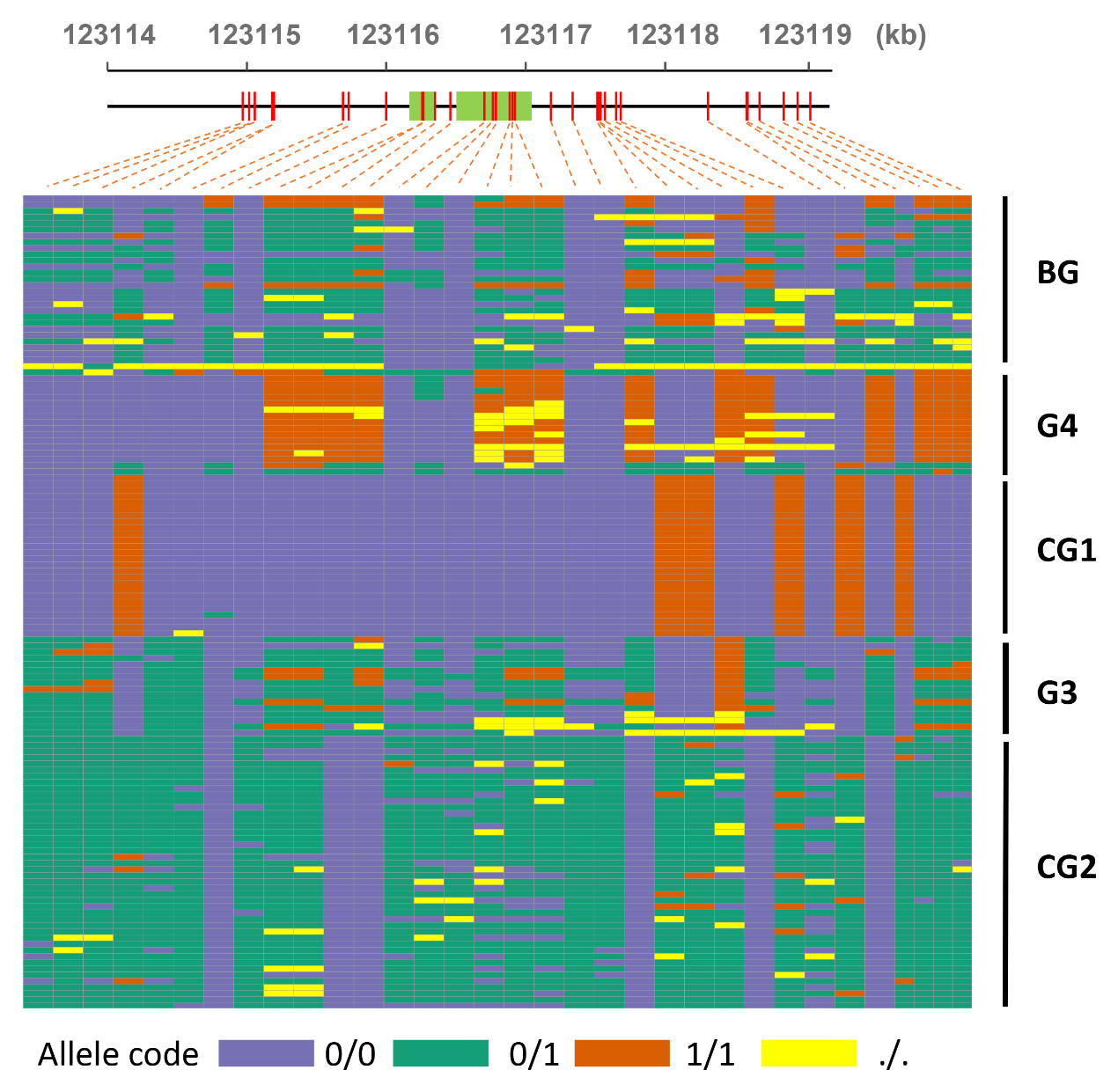


**Fig S10** Genotype in the genic region of *Asa2G00291.1* in 134 investigated accessions of garlic.


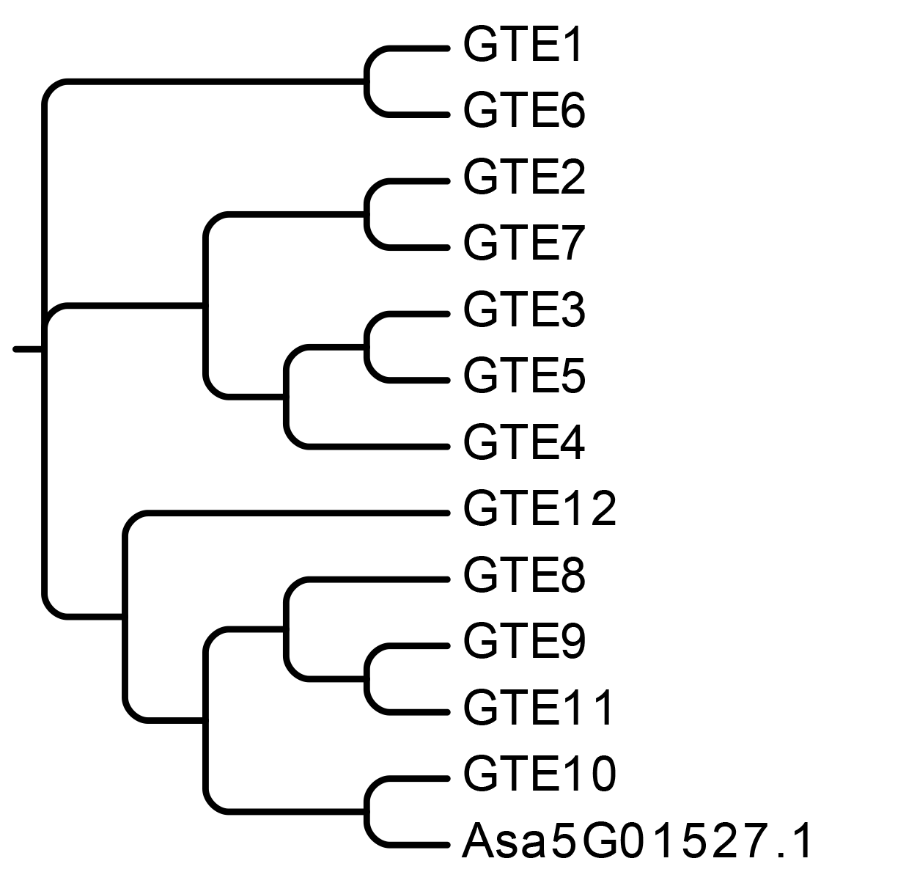


**Fig S11** Phylogenetic tree of Asa5G01527.1 and Arabidopsis GTE proteins.


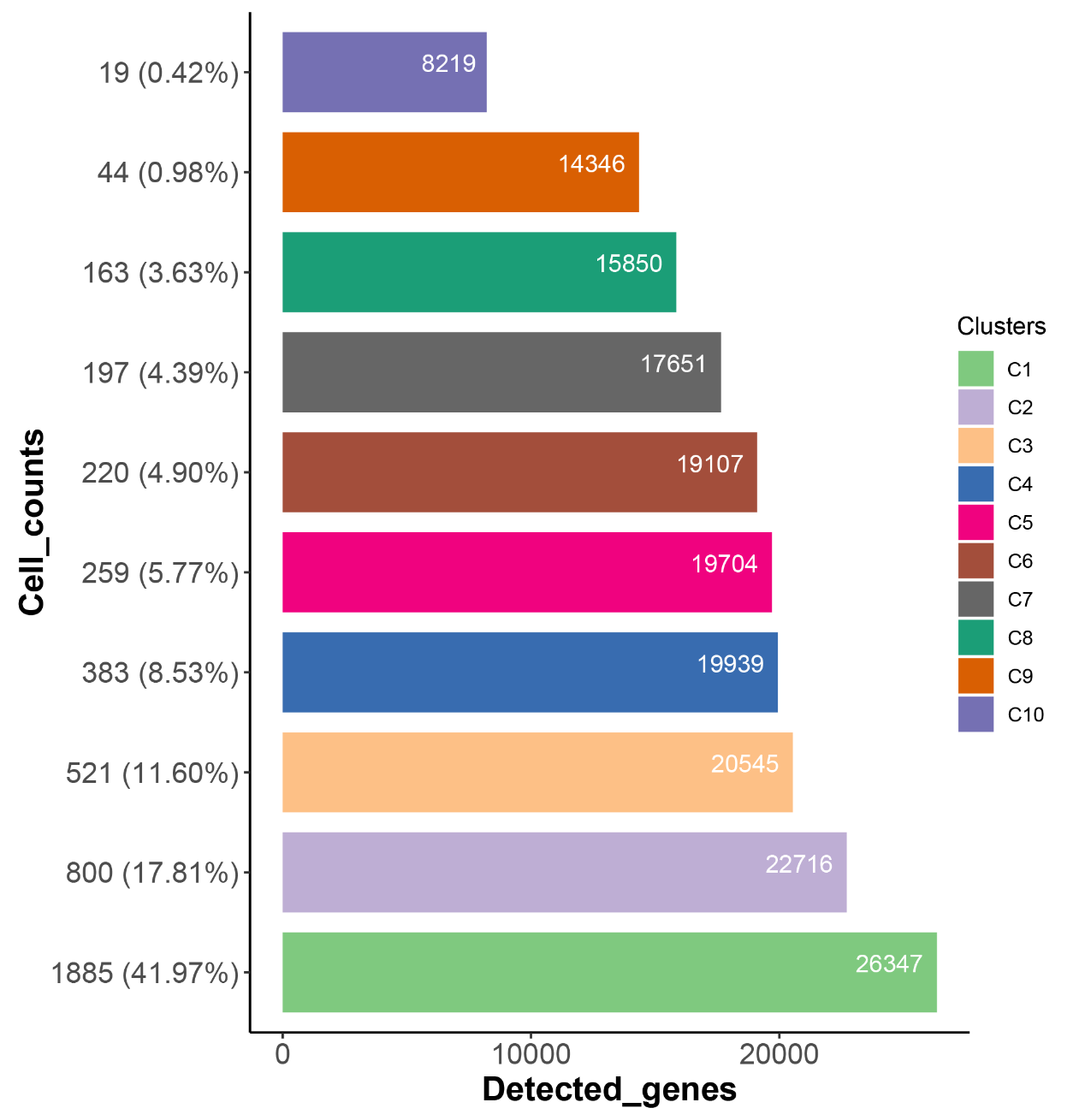


**Fig S12** Cell number, proportion, and active genes detected in each cell cluster from stem base.


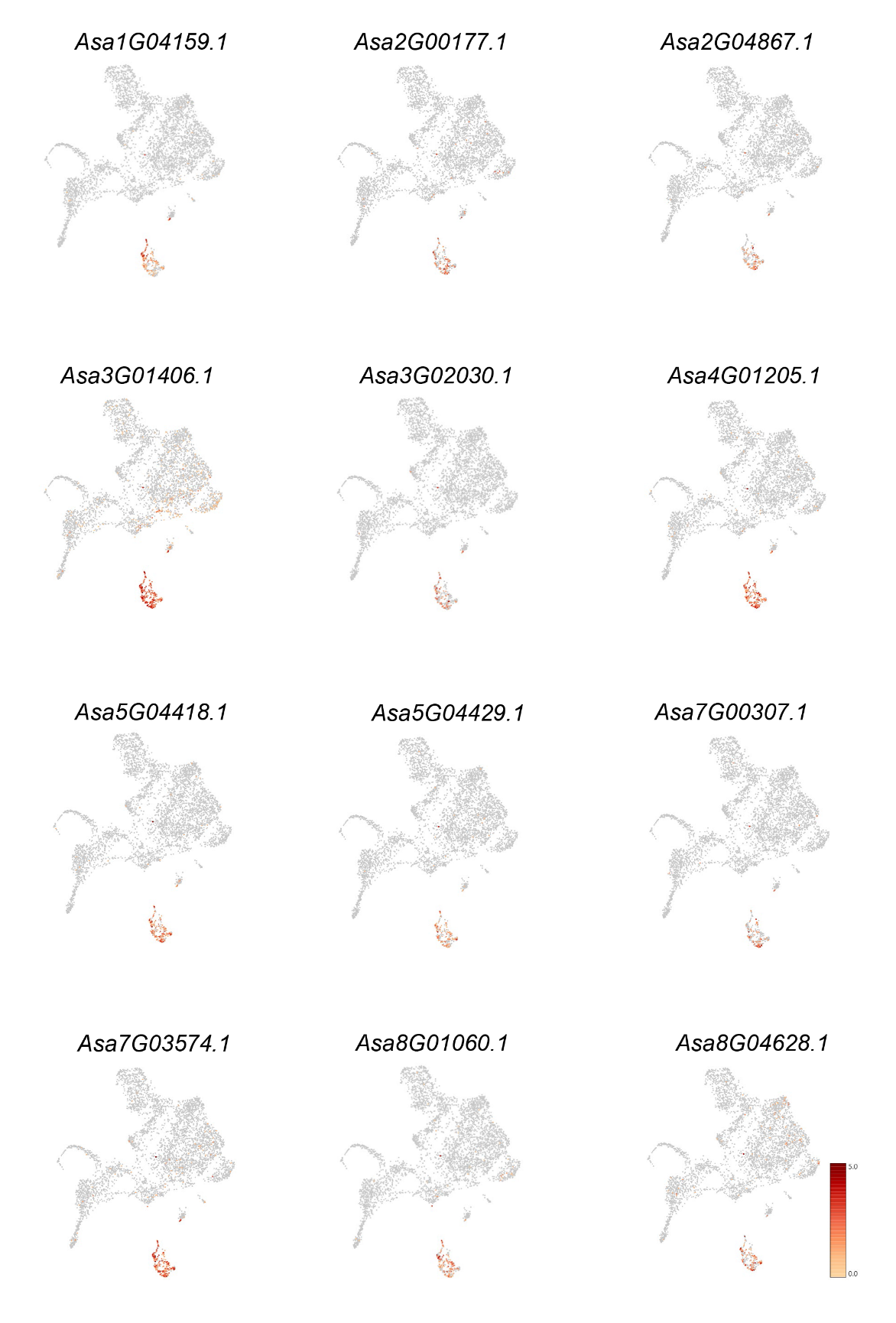


**Fig S13** Expression of twelve garlic genes that encode the homologs of Arabidopsis epidermal cutin and cuticular wax-biosynthetic enzymes.

**
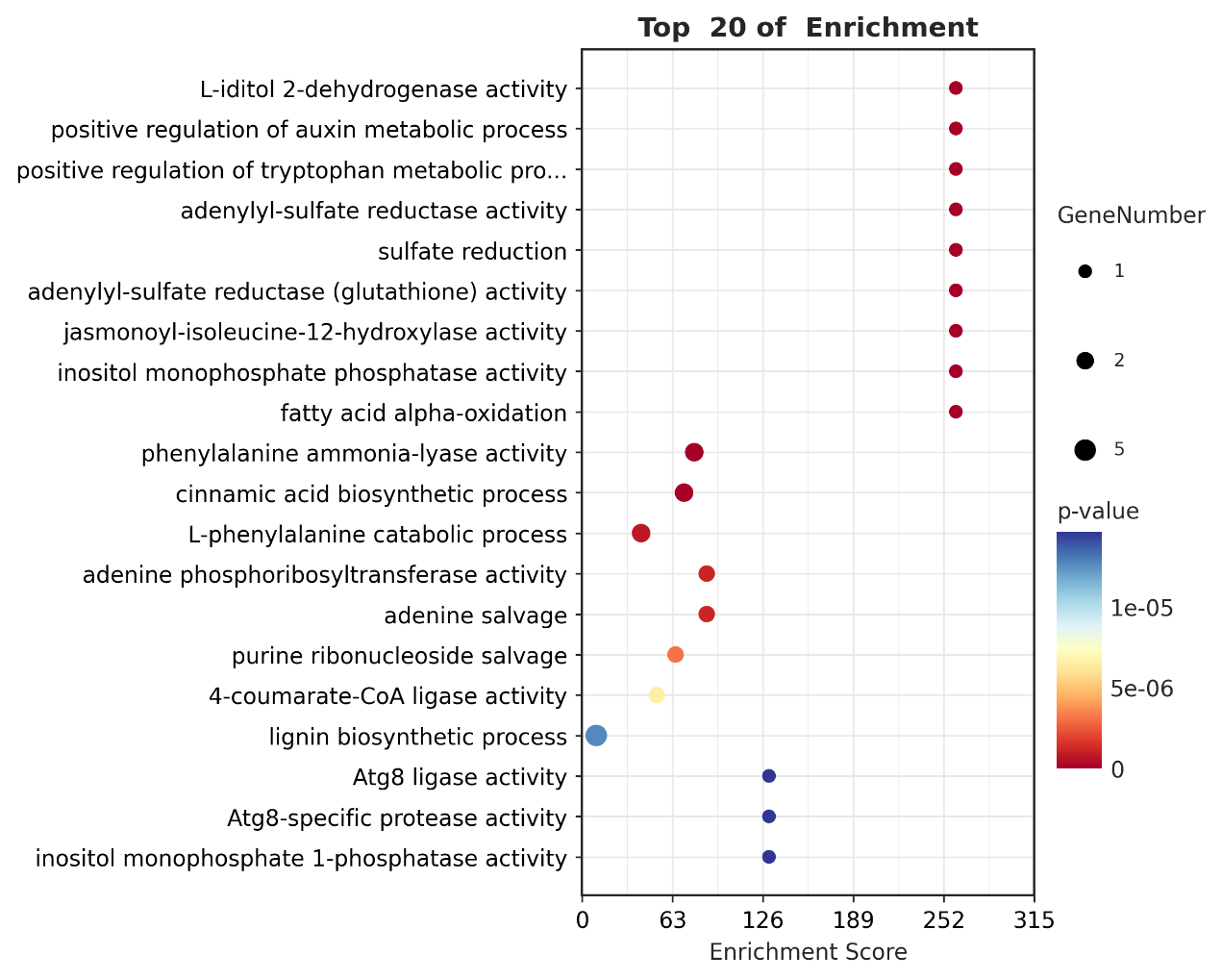
**

**Fig S14** Enrichment of GO terms by the top 200 highly expressed genes of cells in C3.

**
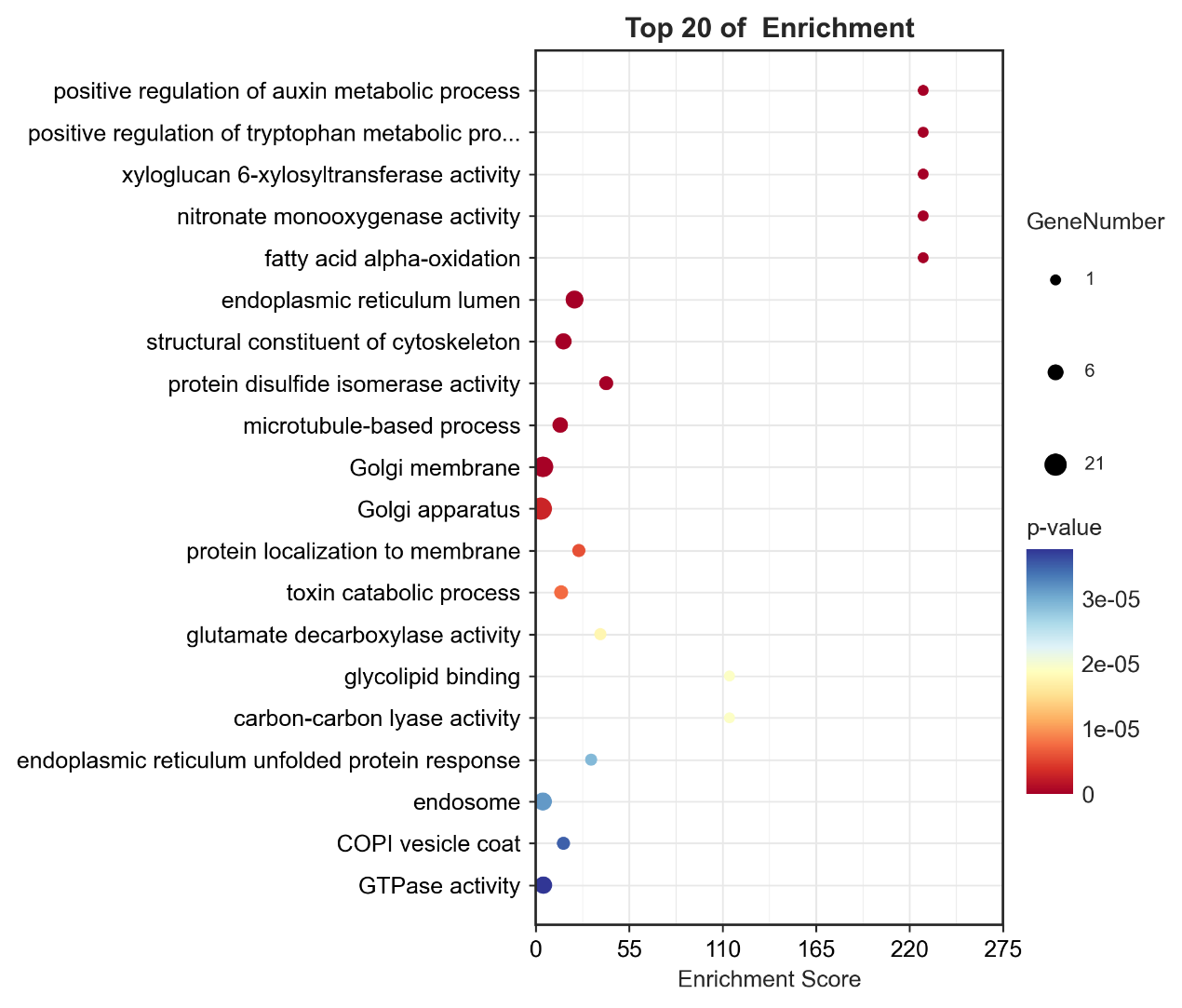
**

**Fig S15** Enrichment of GO terms by the top 200 highly expressed genes of cells in C4.

**
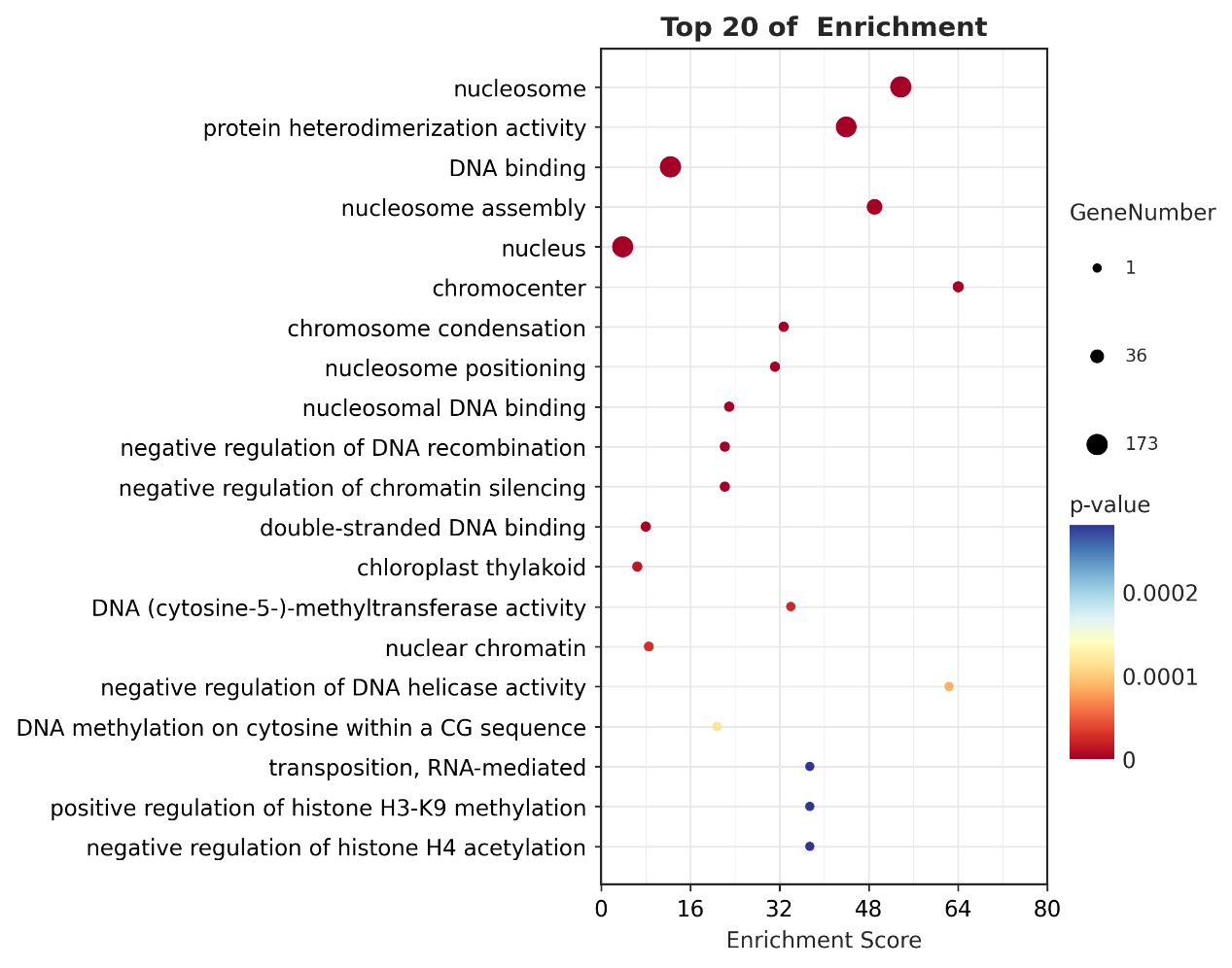
**

**Fig S16** Enrichment of GO terms by the top 200 highly expressed genes of cells in C5.

**
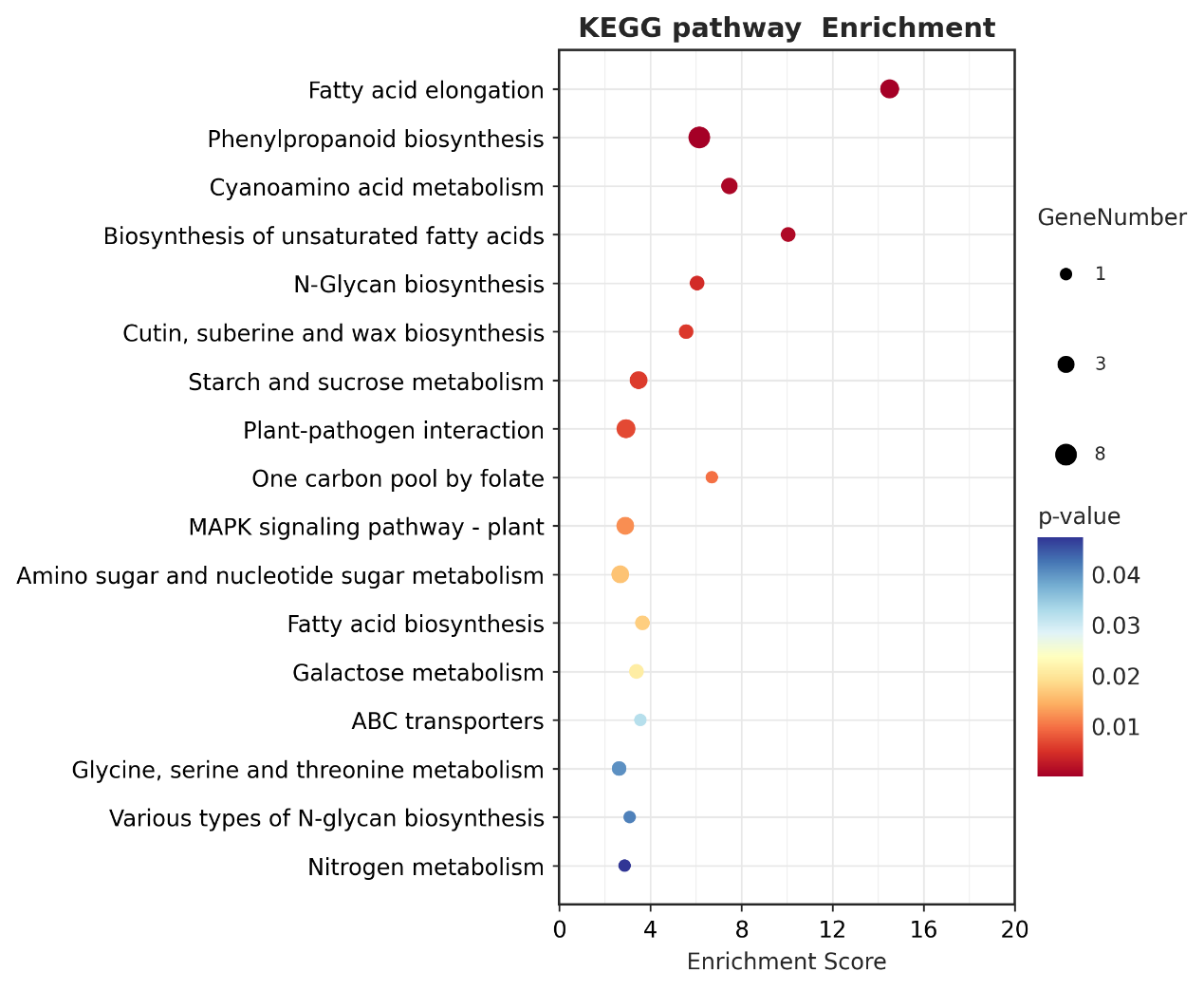
**

**Fig S17** KEGG pathways enriched by the top 200 highly expressed genes of cells in C6.

**
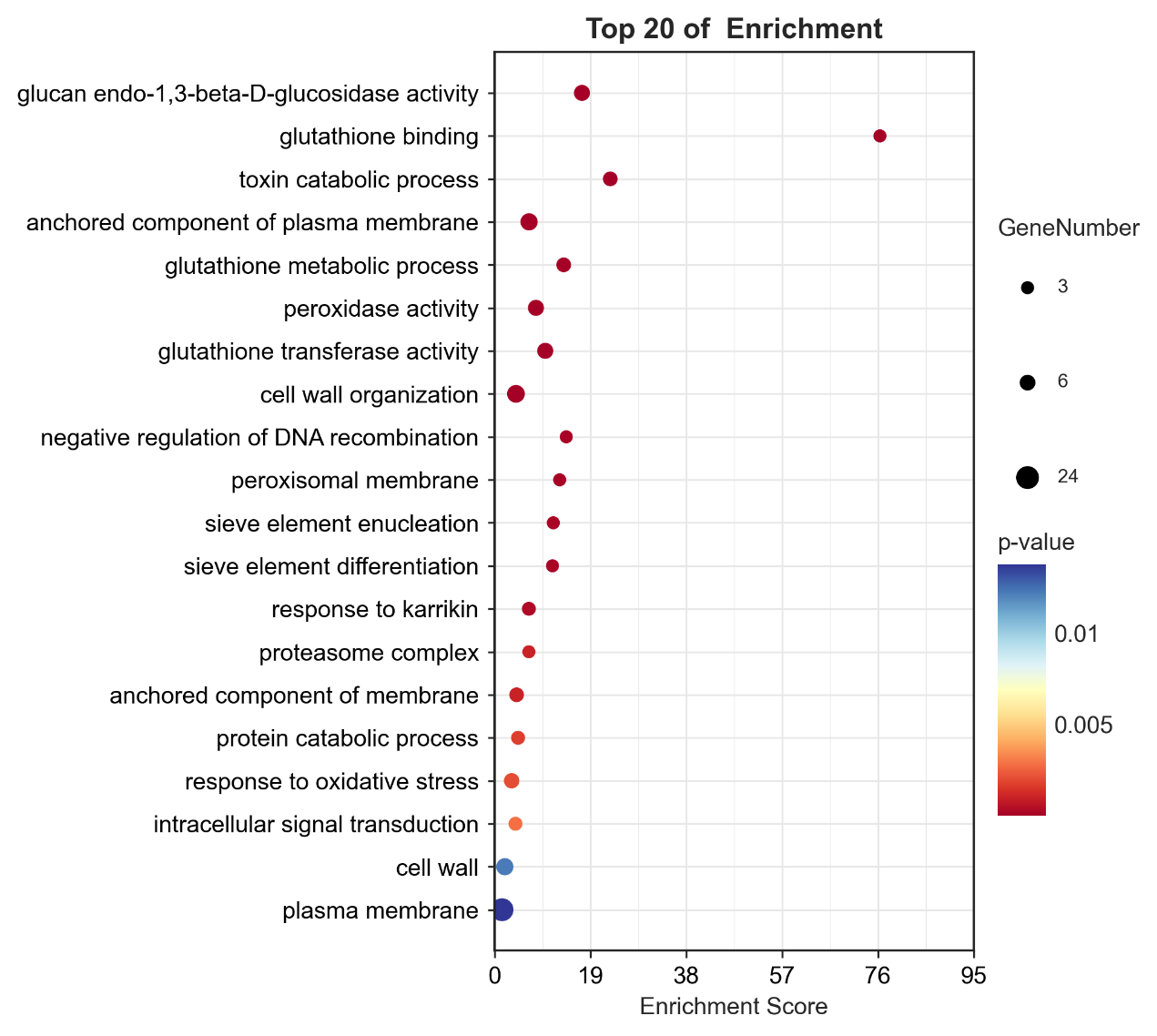
**

**Fig S18** Enrichment of GO terms by the top 200 highly expressed genes of cells in C8.

**
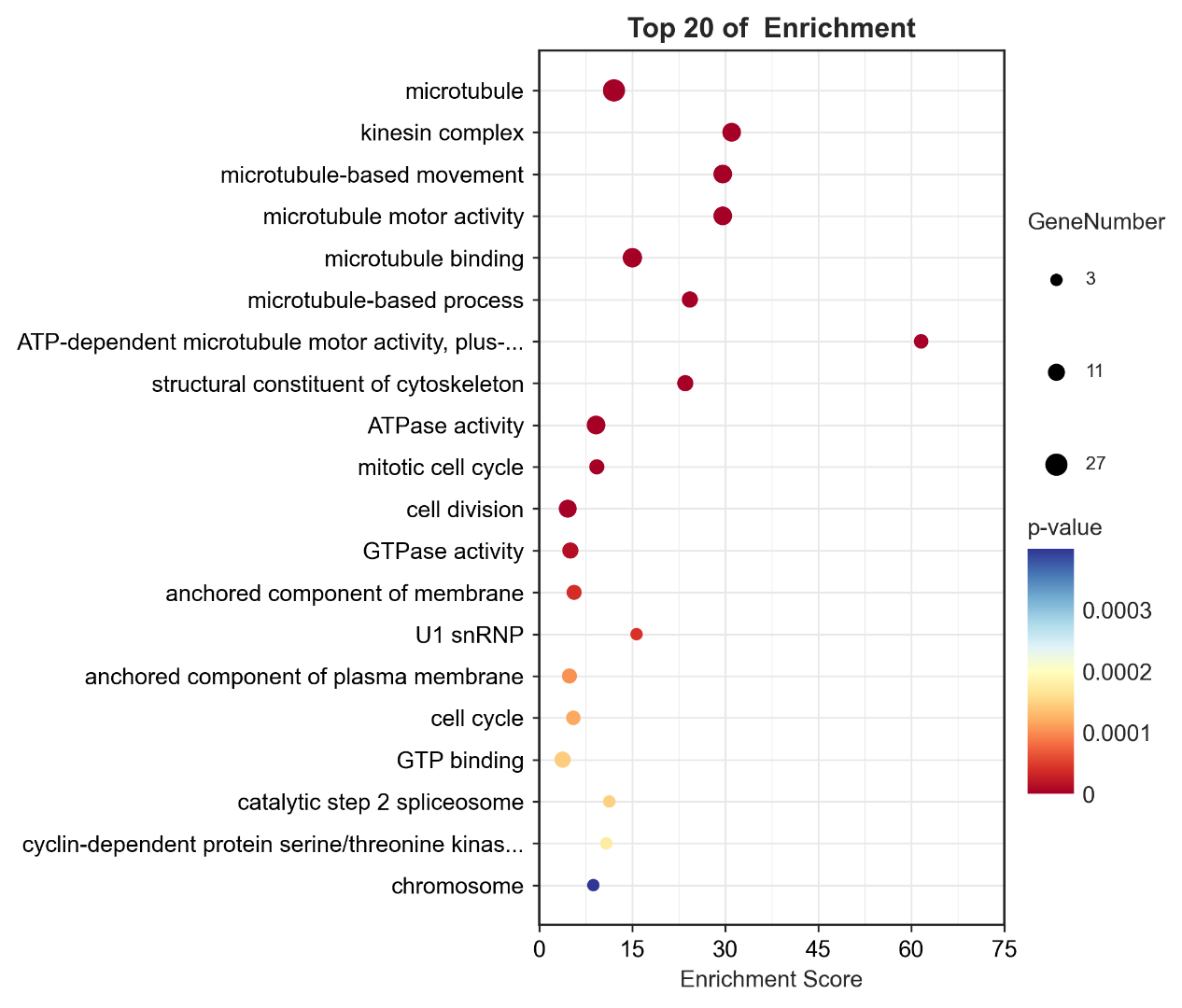
**

**Fig S19** Enrichment of GO terms by the top 200 highly expressed genes of cells in C9.


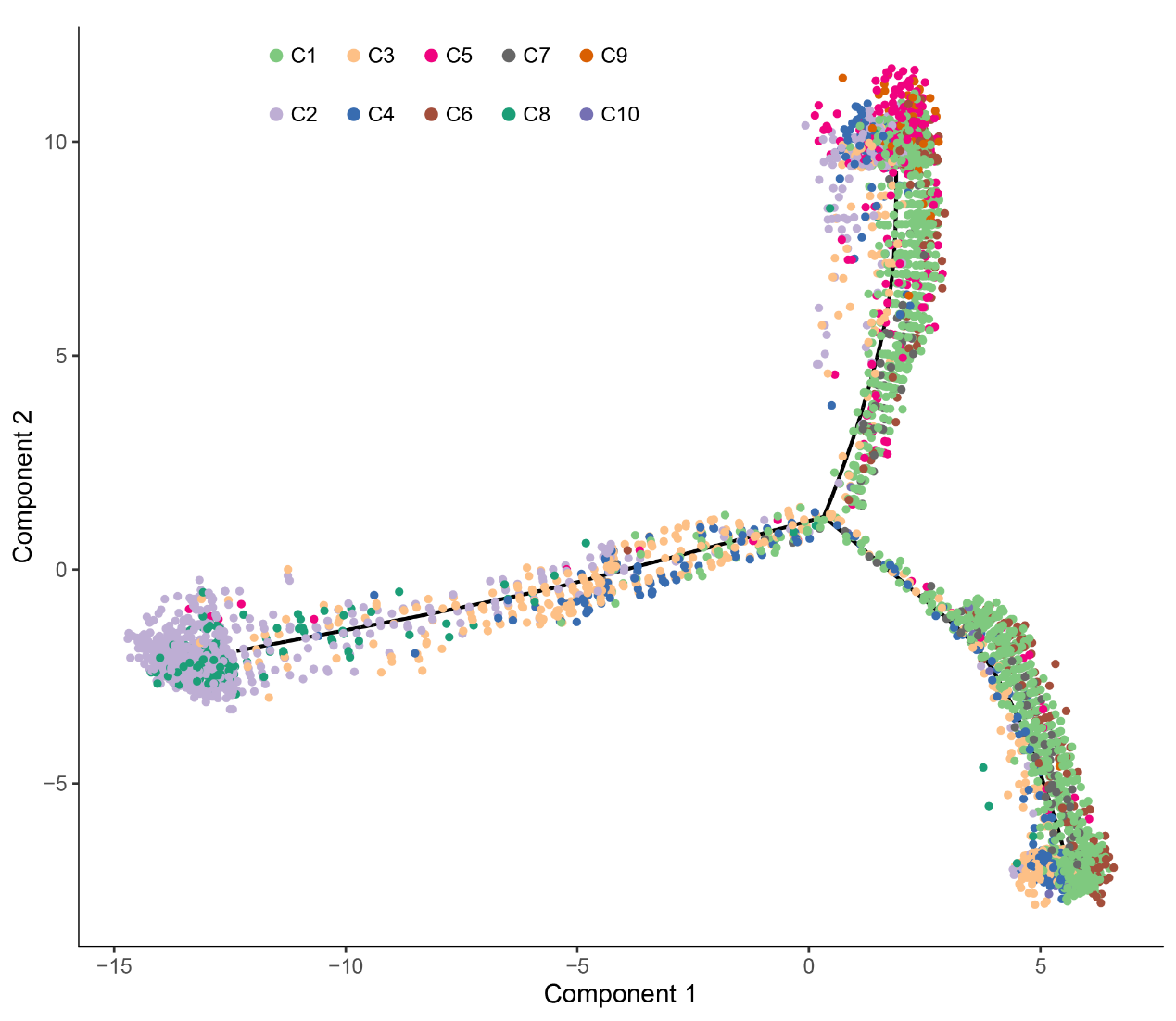


**Fig S20** Trajectory of cells from stem base along pseudo-time. Each dot represents a single cell.


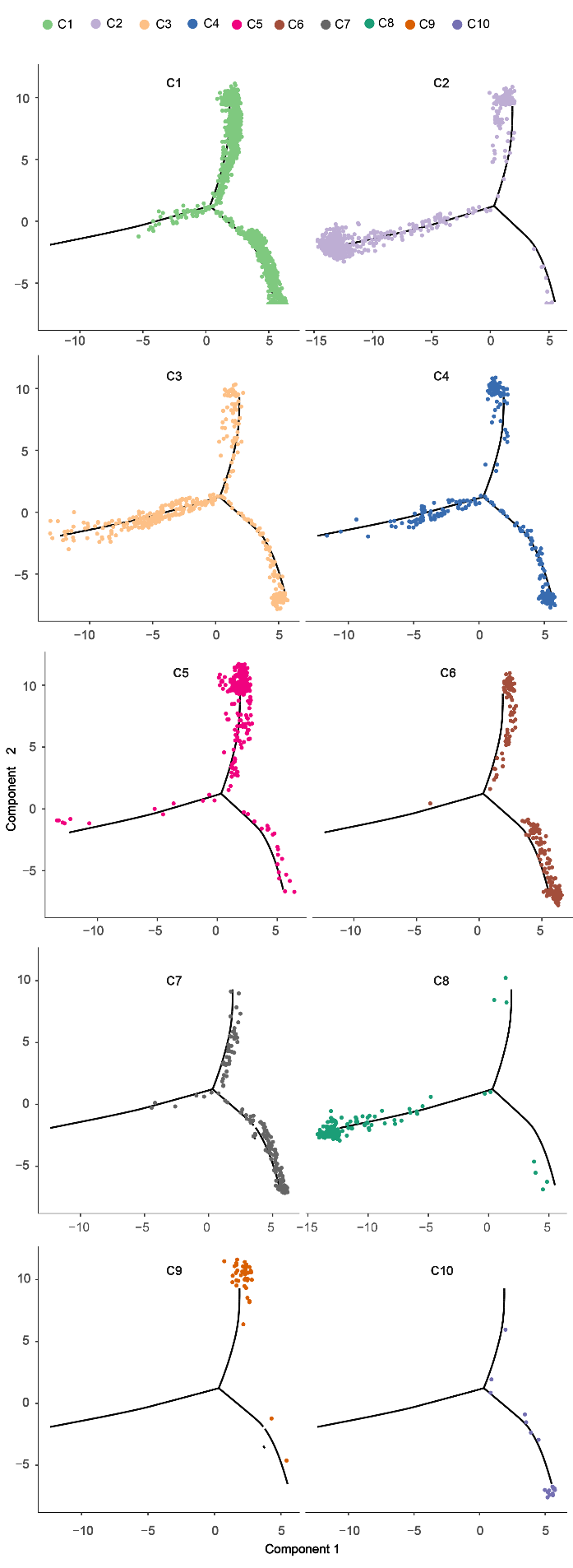


**Fig S21** Pseudo-time trajectory of cells of each cluster in stem base. Each dot represents a single cell.

**Fig S22** Dynamic expression change of *Asa3G03399.1* in bulb part after treating by cold environment.


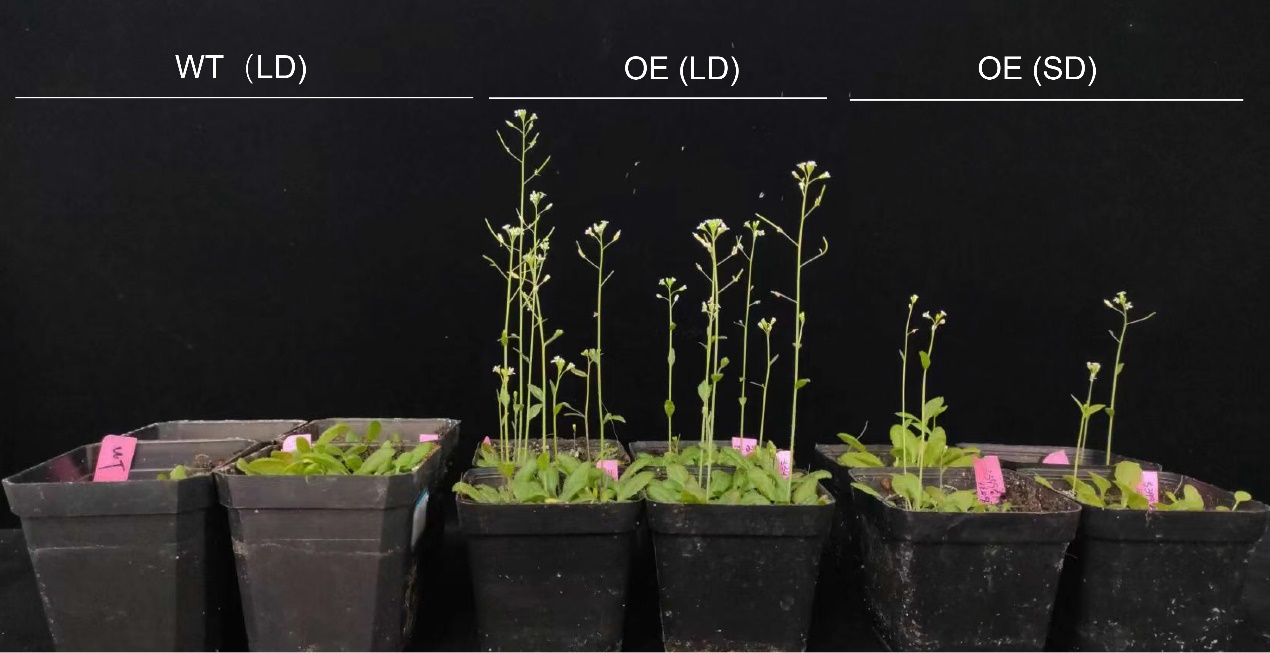


**Fig 23** Comparison of flowering time between wild (WT) and transgenic Arabidopsis (OE) with overexpression of *Asa3G03399.1*. LD and SD indicate that Arabidopsis grows in the conditions of long day (16h daylight/8h dark) and short day (8h daylight/16h dark), respectively.


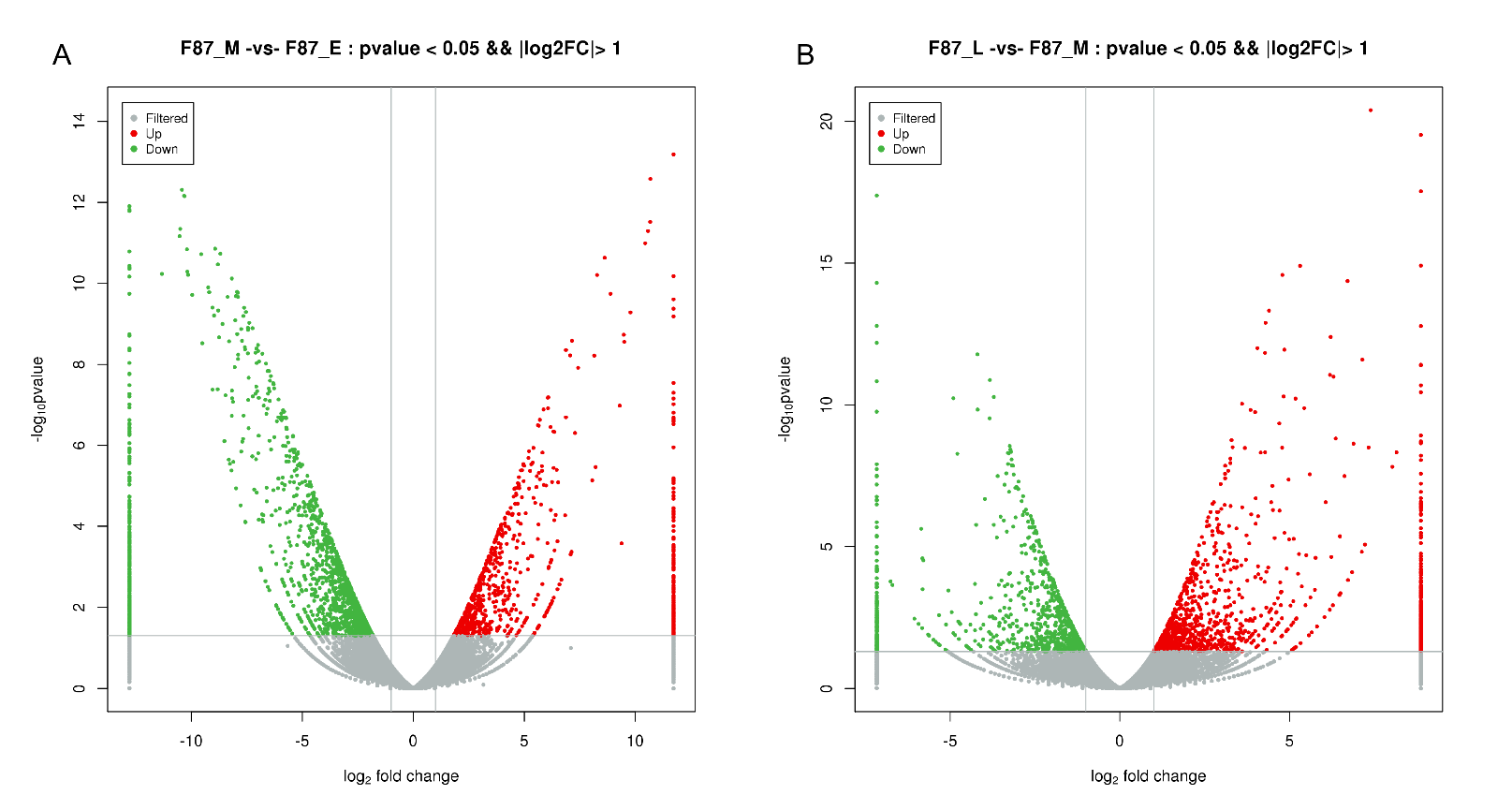
**Fig S24** Genes with expression changes in the garlic flower under middle (A) and late (B) developmental stage. Red and green dots represent genes with an up-regulated and down-regulated expression, and grey dots indicate genes whose expression are not changed significantly.


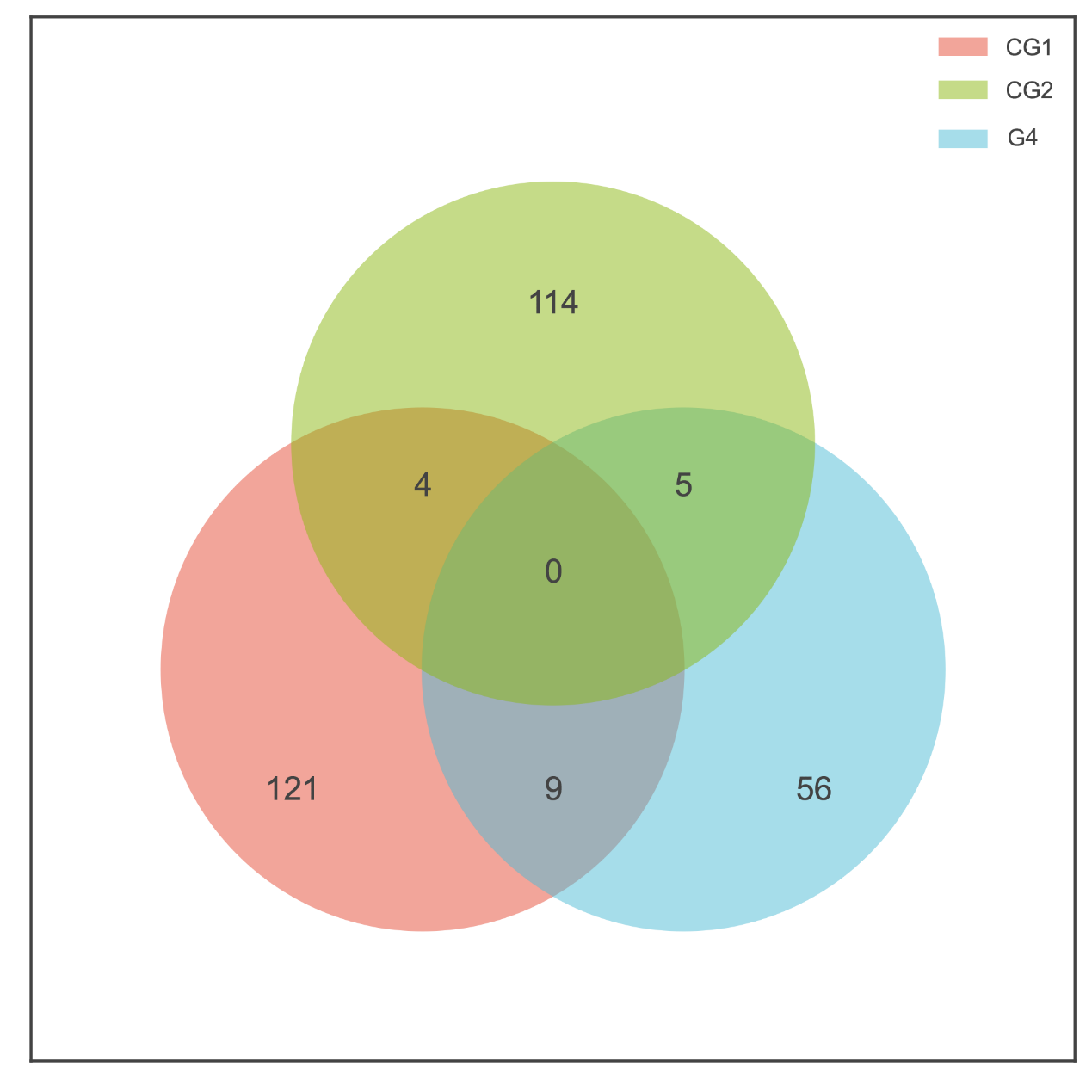


**Fig S25** Venn diagram of flower development-related genes underwent significant selection in CG1, OG, and CG2, respectively.


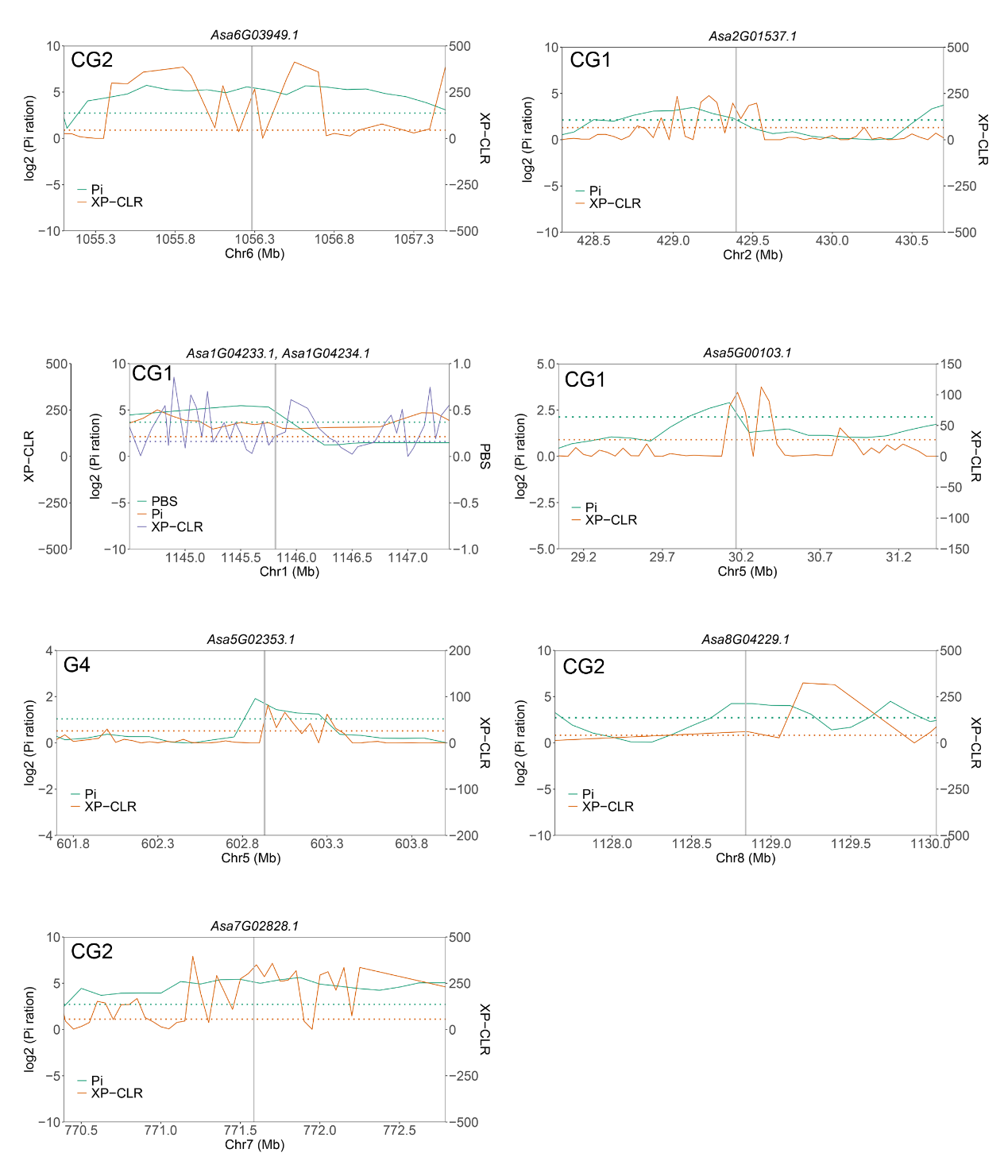


**Fig S26** Distribution of the ratio of nucleotide diversity, XP-CLR and *F_ST_* values in the region near to the flower development-related candidates


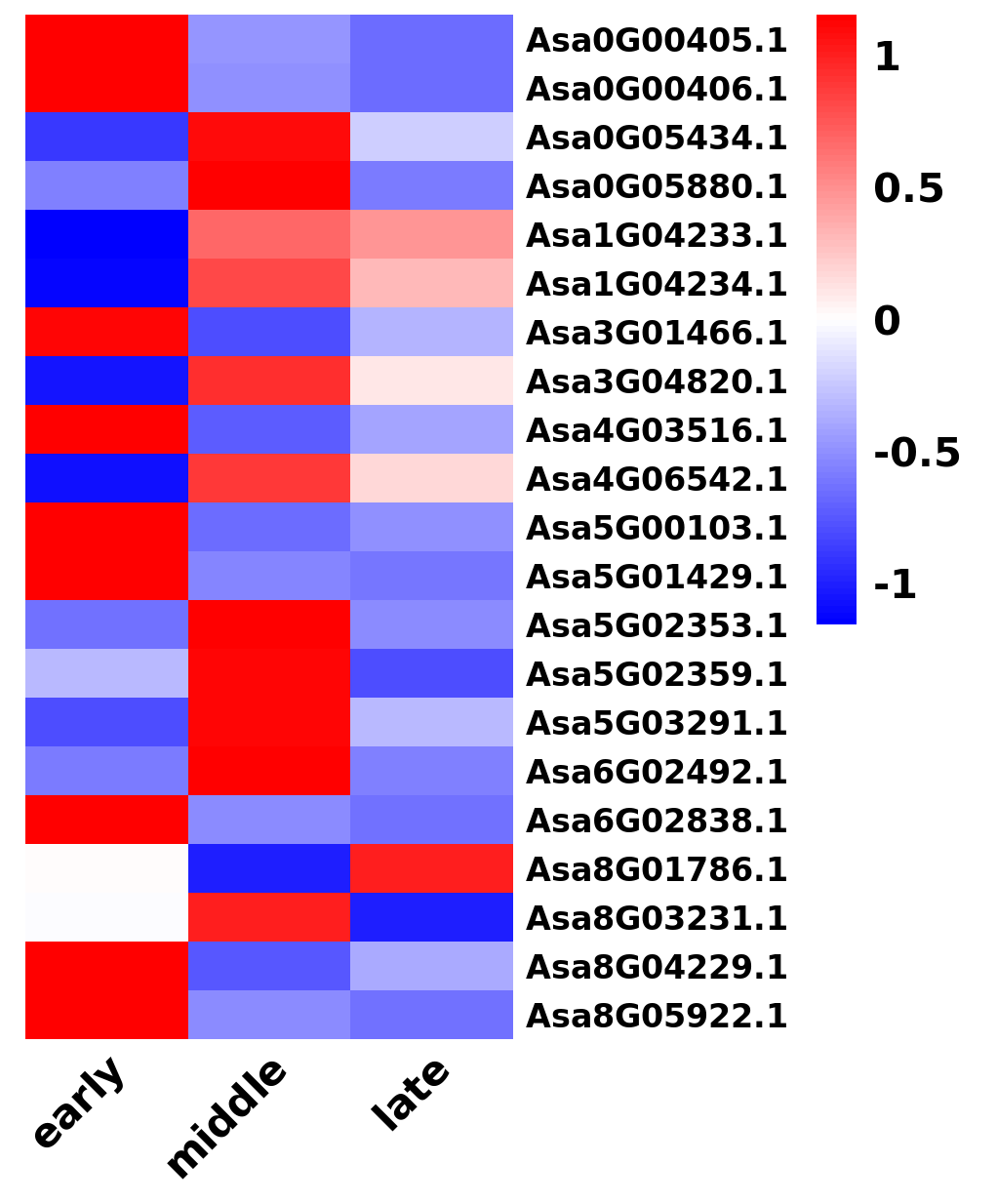


**Fig S27** Garlic NAC genes with expression change during flower-developmental stages.
